# Identification of stable QTLs for vegetative and reproductive traits in the microvine (*Vitis vinifera* L.) using the 18 K Infinium chip

**DOI:** 10.1186/s12870-015-0588-0

**Published:** 2015-08-19

**Authors:** Cléa Houel, Ratthaphon Chatbanyong, Agnès Doligez, Markus Rienth, Serena Foria, Nathalie Luchaire, Catherine Roux, Angélique Adivèze, Gilbert Lopez, Marc Farnos, Anne Pellegrino, Patrice This, Charles Romieu, Laurent Torregrosa

**Affiliations:** Montpellier SupAgro, UMR AGAP, F-34060 Montpellier, France; INRA, UMR AGAP, F-34060 Montpellier, France; Fondation Jean Poupelain, 30 rue Gâte Chien, F-16100 Javrezac, France; Changins, Haute Ecole de Viticulture et Oenologie, 1260 Nyon, Switzerland; Dipartimento di Scienze Agrarie e Ambientali, University of Udine, via delle Scienze 208, I-33100 Udine, Italy; Montpellier SupAgro, UMR LEPSE, F- 34060 Montpellier, France

## Abstract

**Background:**

The increasing temperature associated with climate change impacts grapevine phenology and development with critical effects on grape yield and composition. Plant breeding has the potential to deliver new cultivars with stable yield and quality under warmer climate conditions, but this requires the identification of stable genetic determinants. This study tested the potentialities of the microvine to boost genetics in grapevine. A mapping population of 129 microvines derived from Picovine x Ugni Blanc *flb*, was genotyped with the Illumina® 18 K SNP (Single Nucleotide Polymorphism) chip. Forty-three vegetative and reproductive traits were phenotyped outdoors over four cropping cycles, and a subset of 22 traits over two cropping cycles in growth rooms with two contrasted temperatures, in order to map stable QTLs (Quantitative Trait Loci).

**Results:**

Ten stable QTLs for berry development and quality or leaf area were identified on the parental maps. A new major QTL explaining up to 44 % of total variance of berry weight was identified on chromosome 7 in Ugni Blanc *flb*, and co-localized with QTLs for seed number (up to 76 % total variance), major berry acids at green lag phase (up to 35 %), and other yield components (up to 25 %). In addition, a minor QTL for leaf area was found on chromosome 4 of the same parent. In contrast, only minor QTLs for berry acidity and leaf area could be found as moderately stable in Picovine. None of the transporters recently identified as mutated in low acidity apples or Cucurbits were included in the several hundreds of candidate genes underlying the above berry QTLs, which could be reduced to a few dozen candidate genes when *a priori* pertinent biological functions and organ specific expression were considered.

**Conclusions:**

This study combining the use of microvine and a high throughput genotyping technology was innovative for grapevine genetics. It allowed the identification of 10 stable QTLs, including the first berry acidity QTLs reported so far in a *Vitis vinifera* intra-specific cross. Robustness of a set of QTLs was assessed with respect to temperature variation.

**Electronic supplementary material:**

The online version of this article (doi:10.1186/s12870-015-0588-0) contains supplementary material, which is available to authorized users.

## Background

Climate change is expected to modify several environmental factors, including temperature, CO_2_ concentration, radiation level, water availability, wind speed and air moisture, and to noticeably affect crop production [1]. Air and land temperatures on Earth’s surface are predicted to increase from 1.1 to 6.4 °C by the end of the 21^th^ century [2], in addition to the past temperature rises. Temperature and rainfall are major climatic factors influencing grapevine phenology, yield, berry composition and wine quality [3, 4]. Heat stress is more difficult to cope with than drought stress, which can be mitigated through irrigation or rootstock selection [5]. According to Hannah et al. [6], most of vine growing regions will undergo a global warming of 2 °C to 4 °C in the next decades. Mild to moderate temperature increases (less than +4 °C compared to ambient temperature) were shown to advance grapevine vegetative development and the whole fruit ripening period up to five weeks earlier, *i.e.* at the time of maximum summer temperatures [4, 7, 8]. Phenological changes may negatively impact berry development program and composition. Indeed, warmer climate in the past resulted in higher sugar level and lower contents of organic acids, phenolics and aroma [9–13]. Such alterations of berry composition directly impair the organoleptic quality and the stability of wines [14]. Moreover, high temperature promotes disease development [15], reduces carbohydrate reserves in perennial organs [16], decreases bud fertility, inhibits berry set and, as a result, lowers final yield [17–19].

Negative impacts of climate change on viticulture sustainability and wine quality may be mitigated by: i) viticultural practices such as irrigation or canopy management [20], ii) wine processing like acidification or electro-dialysis, iii) shifting of the vine growing areas towards higher altitude or latitude regions [6, 21, 22] and iv) breeding new cultivars better adapted to the climate changes [23]. The first two methods are widely used, although they are only short-term solutions with limited efficiency. The shift of grape growing areas to cooler climate regions would have dramatic socio-economic consequences. Thus, the development of new cultivars appears to be the best long-term solution for a sustainable viticulture maintaining premium wine production under global warming. However, it requires improving the knowledge on the genetics of key grapevine functions under various environments.

Quantitative Trait Loci (QTLs) repeated over years have been identified in grapevine in usual climate and cultivation conditions. They are notably QTLs for berry size and seedlessness [24, 25], yield components [26], phenology [27, 28], muscat flavour [29, 30], anthocyanin composition [31], tannin composition [32], fruitfulness [33], cluster architecture [34] and disease resistance (*e.g.* [35, 36]). However, no attempts have been made to test their stability regarding large temperature variations. Molecular physiology and genetic studies have increased our knowledge on the regulation of grapevine reproductive development, including flowering [37], berry growth [38, 39], organic acid pathways [40], tannin [41] or anthocyanin accumulation [42, 43] and sugar uploading [44]. The physiological and molecular adaptation of the grapevine to heat stress was recently addressed. Although a slight temperature increase accelerates berry development, high temperatures and/or heat stress (>35 °C) were shown to produce opposite effect, thus delaying berry ripening [4, 17]. Luchaire *et al.* [45] and Rienth *et al.* [46] showed that the carbon flow toward the internodes was dramatically impaired under heat stress, leading to increasing the flowering to ripening time-lag, and to noticeable reprogramming of berry transcriptome.

The genetic control of grapevine adaptation to abiotic stresses remains poorly understood because it requires experimentations on large populations under multi-environment conditions. A few QTLs for water use efficiency and transpiration under duly controlled water stress have been found [47, 48]. Regarding the adaptation to temperature stress, no QTL has yet been identified in grapevine. However, the identification of genetic determinants is critical for the development of temperature-tolerant grapevine cultivars. Furthermore, as for other perennial crops, grapevine breeding is a slow and challenging process in order to combine desirable fruit quality and disease tolerance traits [49]. In grapevine, the breeding process can be noticeably accelerated combining marker-assisted selection [50] and short cycling material such as the microvine [51].

The aim of this work was to identify stable QTLs for a large set of vegetative and reproductive traits in grapevine under contrasted temperature conditions. A pseudo-F1 mapping population of 129 microvine offsprings, derived from a cross between the Picovine [51] and the Ugni Blanc *flb* mutant [52] was genotyped using a 18 K Single Nucleotide Polymorphism (SNP) Illumina® chip and phenotyped for 43 traits over up to nine cropping cycles. Fourteen QTLs for berry development and composition or leaf area were found repeated over at least two conditions, among which 10 were stable over at least half of the environments explored.

## Results

### Phenotypic data

The grapevine population from Picovine 00C001V0008 x Ugni Blanc *flb* (*V. vinifera* L.) was phenotyped in nine experimental conditions for up to 43 vegetative and reproductive traits (Table 1).

**Table 1:** Trait abbreviations and descriptions (units, years and growing conditions)

					Environments								
					Greenhouse	Outdoors				Temperature experiments			
					2011	2011	2012	2013	2014	2013		2014	
		Trait	Abreviation	Method						Hot	Cool	Hot	Cool
Vegetative		Budburst time (cumulated GDD after the 15^th^ of March)	BB	calculated			X	X	X				
		Phyllochron (GDD/leaf)	PHY	calculated	X		X	X		X	X	X	X
		Leaf area (cm^2^/leaf)	LA	calculated	X	X	X	X		X	X	X	X
		Leaf mass per area (mg/cm^2^)	LMA	measured	X	X		X		X	X	X	X
		Internode length (mm)	IL	calculated	X	X	X	X		X	X	X	X
Reproductive		Number of pre-formed inflorescences in winter buds per plant	NBI	measured		X	X	X	X				
	Green lag phase	Position of first pre-formed inflorescence	PBI	measured			X	X	X				
		Period from inflorescence appearance to 50 % flowering (days)	PIF	calculated			X	X					
		Period from 50 % flowering to 50 % *véraison* (days)	PFV	calculated			X	X					
		Berry weight (g)	BWG	measured		X	X	X		X	X	X	X
		Citrate (mEq/kg.FW)	CiG	measured			X	X		X	X		
		Malate (mEq/kg.FW)	MaG	measured		X	X	X		X	X		
		Tartrate (mEq/kg.FW)	TaG	measured		X	X	X		X	X		
		Total acids (mEq/kg.FW)	ToAG	calculated		X	X	X		X	X		
		Malate/tartrate ratio	MTG	calculated		X	X	X		X	X		
		Malate/total acids ratio	MOG	calculated			X	X		X	X		
		Tartrate/total acids ratio	TOG	calculated			X	X		X	X		
		Citrate/total acids ratio	COG	calculated			X	X		X	X		
		Glucose (mM/kg.FW)	GuG	measured			X	X		X	X		
		Fructose (mM/kg.FW)	FuG	calculated			X	X		X	X		
		Total sugars (mM/kg.FW)	ToSG	calculated			X	X		X	X		
		Glucose/fructose ratio	GFG	calculated			X	X		X	X		
		Potassium (mM/kg.FW)	KG	measured	X	X	X	X		X	X		
		Total acids?+?total sugars?+?potassium (mM/Kg.FW)	ASKG	calculated			X	X		X	X		
	Maturity stage	Berry weight (g)	BWM	measured		X	X	X					
		Number of berries per cluster	NB	measured	X	X	X	X		X	X	X	X
		Number of clusters per ten phytomers	NC	measured	X	X	X	X					
		Number of seeds per berry	NS	measured			X	X		X	X		
		Seed weight (mg)	SW	measured			X	X					
		Citrate (mEq/kg.FW)	CiM	measured			X	X					
		Malate (mEq/kg.FW)	MaM	measured			X	X					
		Tartrate (mEq/kg.FW)	TaM	measured			X	X					
		Total acids (mEq/kg.FW)	ToAM	calculated			X	X					
		Malate/tartrate ratio	MTM	calculated			X	X					
		Malate/total acids ratio	MOM	calculated			X	X					
		Tartrate/total acids ratio	TOM	calculated			X	X					
		Citrate/total acids ratio	COM	calculated			X	X					
		Glucose (mM/kg.FW)	GuM	measured			X	X					
		Fructose (mM/kg.FW)	FuM	measured			X	X					
		Total sugars (mM/kg.FW)	ToSM	measured			X	X					
		Glucose/fructose ratio	GFM	calculated			X	X					
		Potassium (mM/kg.FW)	KM	measured			X	X					
		Total acids?+?total sugars?+?potassium (mM/Kg.FW)	ASKM	calculated			X	X					

GDD: growing degree-day

The distributions of phenotypic data in all environments are shown in Additional file 1. Broad sense heritability and the median, maximum and minimum values for each trait are given in Table 2. All traits displayed continuous variation within environments. Seed number per berry was clearly bimodal. Some growth conditions induced very different distributions (Additional file 1), indicating that individuals displayed different plasticity of studied traits to environmental changes (mainly temperature) within the population. This was particularly true tartrate ratio/tartrate ratio. For most phenotypes, the population showed a large segregation of the phenotypes, *e.g.*: phyllochron (PHY; 15 to 120 GDD/leaf), leaf area (LA; 10 to 290 cm^2^/leaf), number of pre-formed inflorescences in winter buds per plant (NBI; 0.25 to 3.8), number of berries per cluster (NB; 5 to 75), berry weight at green lag phase (BWG; 0.2 to 2.2 g), berry weight at maturity (BWM; 0.5 to 3.2 g), total berry acidity at green lag phase (ToAG; 220 to 780 mEq/kg.FW), malate/tartrate ratio at green lag phase (MTG; 0.75 to 5.2), total sugars at green lag phase (ToSG; 5 to 120 mM/kg.FW), total sugars at maturity (ToSM; 350 to 1200 mM/kg.FW), potassium content at green lag phase (KG; 15 to 120 mM/kg.FW).

**Table 2:** Minimum, median, maximum and broad-sense heritability values for each trait

Vegetative traits								
	BB	PHY	LA	LMA	IL			
*H* ^2^	**0.46**	0.16	0.18	0.05	0.27			
Minimun	9	16	17	2	5			
Median	30	27	118	4	21			
Maximum	153	118	308	12	39			
Inflorescence traits								
	NBI	PBI	PIF	PFV				
*H* ^2^	0.02	0.27	0.30	**0.40**				
Minimun	0.1	3	18	48				
Median	1.5	6	21	56				
Maximum	4.0	8	25	73				
Berry traits								
	NC	NB	BWG	BWM	NS	SW		
*H* ^*2*^	**0.47**	0.28	**0.52**	**0.67**	**0.80**	**0.43**		
Minimun	0.1	3	0.2	0.5	0.9	27		
Median	2.9	19	1.0	1.3	2.6	46		
Maximum	4.6	86	2.2	3.2	4.0	69		
Berry acid content traits								
At green lag phase	MaG	TaG	CiG	MOG	TOG	COG	MTG	ToAG
*H* ^*2*^	0.20	0.32	0.32	**0.51**	**0.43**	**0.42**	0.39	0.17
Minimun	113	80	1	0.36	0.20	0.001	0.5	241
Median	334	166	7	0.62	0.36	0.015	2.1	509
Maximum	627	260	20	0.80	0.62	0.035	2.5	784
At maturity	MaM	TaM	CiM	MOM	TOM	COM	MTM	ToAM
*H* ^*2*^	0.19	0.13	0.33	0.31	**0.42**	**0.42**	0.34	0.05
Minimun	23	47	1	0.10	0.2	0.010	0.2	93
Median	82	116	5	0.40	0.6	0.027	0.7	197
Maximum	204	210	13	0.70	1.0	0.059	1.7	365
Berry sugar and potassium content traits								
At green lag phase	GuG	FuG	GFG	ToSG	KG	ASKG		
*H* ^*2*^	0.28	0.13	0.24	0.22	0.09	0.01		
Minimun	1	1	0.1	1	16	105		
Median	18	15	1.2	41	53	361		
Maximum	71	132	3.9	177	130	564		
At maturity	GuM	FuM	GFM	ToSM	KM	ASKM		
*H* ^*2*^	0.23	0.16	0.01	0.19	0.23	0.17		
Minimun	125	178	0.7	303	53	458		
Median	420	461	0.9	879	87	1073		
Maximum	647	516	1.1	1365	128	1612		

Bold setting indicates *H* ^2^ ≥ 0.4

**Fig. 1:**
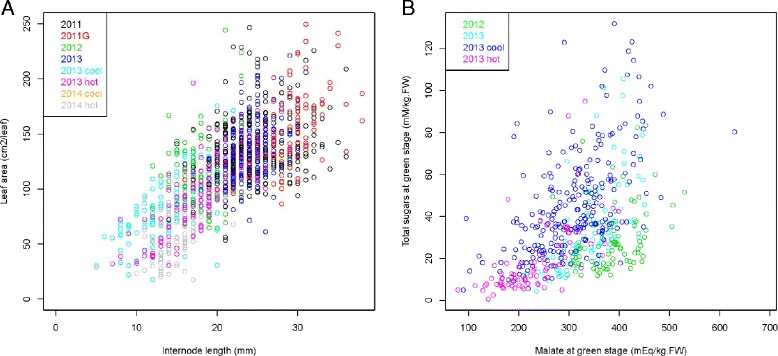
Biplots of vegetative or berry composition related traits in a microvine population. **a**. Leaf area *vs* internode length. **b**. Total sugars *vs* malate concentration at green lag phase

For each environment, all 43 traits were classified according to the Ward hierarchical classification in order to assess correlations between them (Additional file 2). Berry weight at green and maturity stages (BWG, BWM) remained highly correlated regardless of the environment and this also was found true for the correlation between leaf area (LA) and internode length (IL) (Fig. 1a). Moreover, tartrate concentration and tartrate/total acid ratio at green lag phase (TaG, TOG) were correlated to each other and also linked with the number of berries and clusters (NB, NC). However TaG was not related to malate concentration (MaG), which correlated with sugar concentration traits at green lag phase (Fig. 1b).

Seventeen of the 43 phenotyped traits showed correlations (*r* ≥ 0.6) between at least two environments (Additional file 3), but only the number of seeds showed such correlations between all environments.

Most of the models selected to estimate heritability included the environment effect (data not shown). Broad sense heritability (*H*^2^) of the inter-environment genotypic means varied from 0.01 to 0.80 (Table 2), and it was higher than 0.40 for 12 traits out of 43. The number of seeds per berry and berry weight at green lag phase and maturity displayed the highest heritabilities (0.80, 0.67 and 0.52, respectively).

### Genetic maps

Out of the 18 K SNPs on the chip, 6,000 were polymorphic in this population and yielded good quality genotyping data. A subset of these SNPs was selected to build a framework map for each parent suitable for initial QTL detection, with a marker density appropriate for this population size.

The paternal genetic map (Ugni Blanc *flb*; Additional file 4 part A) consisted of 714 SNP markers (of segregation type aaxab only) mapped on 19 linkage groups and covering a total of 1,301 cM. Coverage was mostly satisfying with an average distance of 1.8 cM between adjacent markers and 302 kb/cM. However, some LG parts were not covered, mainly due to the discarding of monomorphic markers (55 % of all initial markers; Additional file 5). It was not due to the absence of markers on the 18 K chip in these regions, since there was no distance between adjacent markers larger than 0.5 Mb on this chip (A. Launay, personnal communication). In a few map gaps however, only non-*vinifera* markers had been defined on the chip, which may not have amplified on this *V. vinifera* population. In two specific regions of LGs 2 and 18, harboring the sex and *Flb* loci, respectively [38, 53], there was simply no male segregation in the population, since the Picovine was homozygous and Ugni Blanc *flb* heterozygous at both these loci and only hermaphrodite offspring with no fleshless berries were retained for this study. All markers from paternal LG 2, on each side of the selected region, exhibited high segregation distortion.

The maternal genetic map (Picovine 00C001V0008; Additional file 4 part B) consisted of 408 SNP markers (353 of type abxaa and 55 of type abxab) mapped on 18 linkage groups spanning a total of 606 cM, with an average inter-marker distance of 1.5 cM and 390 kb/cM. Compared to the paternal map, the number of markers and genome coverage in the maternal map were halved, resulting in a smaller map with markers not covering the entire genome. Picovine comes from a self-fertilization of a microvine [51]. Thus, it is highly homozygous (54 %; MR Thomas, personal communication). LG 7 was even totally missing in the maternal map. Nevertheless, a good colinearity was found between the order of genetic markers and their physical localisation on the genome, in both maps (Additional file 6).

### QTL detection

**Fig. 2:**
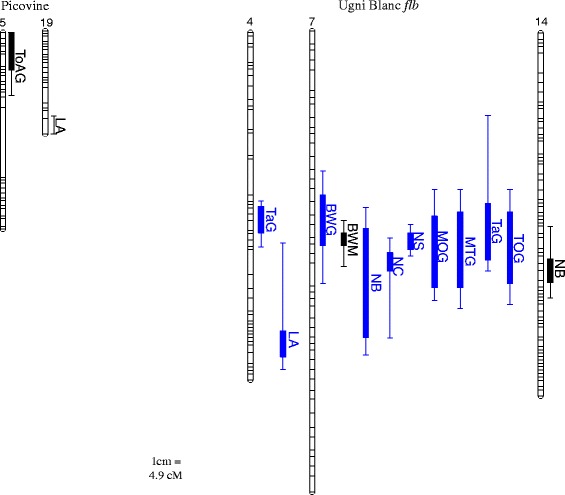
Localisation on the parental genetic maps of a microvine population, of QTLs repeated in at least two different conditions. Stable QTLs, found in at least half of the explored conditions, are displayed in blue. Bars indicate the maximum and minimum value of LOD-1 confidence intervals from QTLs for the same traits identified under at least two environments. Black boxes represent the range of peak LOD values over the different environments. Distances are in Kosambi cM. BWG: Berry weight at green lag phase; BWM: Berry weight at maturity; LA: Leaf area; MOG: Malate/total acids ratio at green lag phase; MTG: Malate/tartrate ratio at green lag phase; NB: Number of berries per cluster at maturity; NC: Number of clusters per ten phytomers at maturity; NS: Number of seeds per berry at maturity; TaG: Tartrate at green lag phase; ToAG: Total acids at green lag phase; TOG: Tartrate/total acids ratio at green lag phase

A hundred and fourteen significant QTLs were identified on parental maps (Additional file 7). Among them, 14 were detected under two environments or more. In this study, a focus was placed on these repeated QTLs only (Table 3; Fig. 2). These QTLs concerned 11 out of the 43 phenotyped traits and were related to leaf area and berry trait variations. Ten of these QTLs were considered as stable since they were detected in at least half of the conditions explored. No repeated QTLxQTL interaction was found.

**Table 3:** Statistically significant repeated QTLs, identified under at least two different growing conditions

Trait	Year	Growing condition^a^	Genetic map	Linkage group	QTL peak position (cM)	Interval position (cM)		LOD	% of variance
**LA**	**2012**	**outdoors**	**Ugni blanc flb**	**4**	**70.9**	*50*	*80.1*	**3**	**10**
**LA**	**2011**	**outdoors**	**Ugni blanc flb**	**4**	**72.4**	**69.3**	**80.1**	**3.6**	**17**
**LA**	**2014**	**hot**	**Ugni blanc flb**	**4**	**72.4**	**69.3**	**77**	**4.8**	**16**
**LA**	**2011**	**greenhouse**	**Ugni blanc flb**	**4**	**77.1**	**69**	**80.1**	**5.1**	**14**
LA	2013	hot	Picovine	19	30.9	26.2	30.9	2.9	10
LA	2014	hot	Picovine	19	30.9	*25.5*	*30.9*	3.5	12
**BWG**	**2013**	**hot**	**Ugni blanc flb**	**7**	**39.0**	*33.3*	**49**	**4.7**	**25**
**BWG**	**2014**	**cool**	**Ugni blanc flb**	**7**	**46.0**	**43**	**49.6**	**13.1**	**43**
**BWG**	**2013**	**cool**	**Ugni blanc flb**	**7**	**46.5**	**44.2**	**48**	**7**	**37**
**BWG**	**2013**	**outdoors**	**Ugni blanc flb**	**7**	**48.0**	**45**	**52**	**7.4**	**26**
**BWG**	**2014**	**hot**	**Ugni blanc flb**	**7**	**48.0**	**45**	**53**	**12.1**	**44**
**BWG**	**2011**	**outdoors**	**Ugni blanc flb**	**7**	**50.0**	**45**	**53.5**	**6.6**	**33**
**BWG**	**2012**	**outdoors**	**Ugni blanc flb**	**7**	**51.0**	**46.5**	*60*	**6.6**	**28**
BWM	2011	outdoors	Ugni blanc flb	7	48.0	*45*	53	9.9	42
BWM	2013	outdoors	Ugni blanc flb	7	51.0	47	*56*	7.6	17
**NB**	**2014**	**hot**	**Ugni blanc flb**	**7**	**47.0**	*42*	**66**	**4.9**	**20**
**NB**	**2014**	**cool**	**Ugni blanc flb**	**7**	**51.2**	**42**	**64.4**	**4.7**	**18**
**NB**	**2011**	**outdoors**	**Ugni blanc flb**	**7**	**72.9**	**61**	**75**	**5**	**24**
**NB**	**2013**	**outdoors**	**Ugni blanc flb**	**7**	**72.9**	**68**	*77*	**5.6**	**18**
NB	2013	hot	Ugni blanc flb	14	53.7	*46*	59.4	3.2	18
NB	2013	outdoors	Ugni blanc flb	14	59.3	55.6	*63*	4.3	13
**NC**	**2012**	**outdoors**	**Ugni blanc flb**	**7**	**52.7**	**49.2**	**54.4**	**4.1**	**20**
**NC**	**2013**	**outdoors**	**Ugni blanc flb**	**7**	**52.7**	*49.2*	*73*	**3.3**	**13**
**NC**	**2011**	**outdoors**	**Ugni blanc flb**	**7**	**57.1**	**51**	**63**	**5.2**	**25**
**NS**	**2013**	**hot**	**Ugni blanc flb**	**7**	**48.0**	*46*	**51**	**16.2**	**63**
**NS**	**2013**	**cool**	**Ugni blanc flb**	**7**	**49.0**	**46**	**53**	**9.9**	**48**
**NS**	**2013**	**outdoors**	**Ugni blanc flb**	**7**	**51.0**	**50**	**52.7**	**35.2**	**76**
**NS**	**2012**	**outdoors**	**Ugni blanc flb**	**7**	**52.0**	**50**	*53.5*	**25.3**	**71**
ToAG	2012	outdoors	Picovine	5	11.3	0	17.8	3.4	6
ToAG	2013	outdoors	Picovine	5	0	*0*	*18.8*	3.1	12
**TaG**	**2013**	**hot**	**Ugni blanc flb**	**4**	**41.3**	*40*	**45**	**7.6**	**31**
**TaG**	**2011**	**outdoors**	**Ugni blanc flb**	**4**	**41.5**	**41**	*49*	**7.6**	**33**
**TaG**	**2012**	**outdoors**	**Ugni blanc flb**	**4**	**47.6**	**44.1**	**51**	**3.8**	**12**
**TaG**	**2013**	**cool**	**Ugni blanc flb**	**7**	**41.0**	*20.1*	**51**	**7**	**35**
**TaG**	**2013**	**hot**	**Ugni blanc flb**	**7**	**42.0**	**32**	**49**	**5.3**	**20**
**TaG**	**2013**	**outdoors**	**Ugni blanc flb**	**7**	**49.2**	**35.1**	**52.7**	**3**	**12**
**TaG**	**2012**	**outdoors**	**Ugni blanc flb**	**7**	**54.4**	**44**	*57*	**8.1**	**29**
**TOG**	**2013**	**cool**	**Ugni blanc flb**	**7**	**43.0**	*37.7*	**49**	**5**	**30**
**TOG**	**2013**	**outdoors**	**Ugni blanc flb**	**7**	**49.0**	**43**	**52**	**3.8**	**14**
**TOG**	**2013**	**hot**	**Ugni blanc flb**	**7**	**60.0**	**47**	*65*	**4.3**	**25**
**MOG**	**2013**	**cool**	**Ugni blanc flb**	**7**	**44.0**	*37.7*	**49**	**4.8**	**30**
**MOG**	**2013**	**outdoors**	**Ugni blanc flb**	**7**	**49.0**	**43**	**52**	**3.5**	**14**
**MOG**	**2013**	**hot**	**Ugni blanc flb**	**7**	**61.0**	**48**	*64.1*	**4.3**	**25**
**MTG**	**2013**	**cool**	**Ugni blanc flb**	**7**	**43.0**	*37.7*	**49**	**5.1**	**31**
**MTG**	**2013**	**outdoors**	**Ugni blanc flb**	**7**	**49.0**	**43**	**52**	**3.3**	**13**
**MTG**	**2012**	**outdoors**	**Ugni blanc flb**	**7**	**54.4**	**49.2**	**57**	**7.6**	**32**
**MTG**	**2011**	**outdoors**	**Ugni blanc flb**	**7**	**57.1**	**42**	*66*	**4.7**	**25**
**MTG**	**2013**	**hot**	**Ugni blanc flb**	**7**	**61.0**	**47**	**65**	**4.3**	**25**

Italic setting indicates the maximum and minimum limits of QTL confidence intervals for a given trait identified under different environments

The stable QTLs, identified in at least half of the environments studied, are displayed in bold

^a^hot and cool growth conditions correspond to the two conditions in controlled growth rooms during the thermal stress experiment

#### Leaf area

Two repeated QTLs explaining up to 12 % and 17 % of leaf area variation were found on Picovine LG 19 and on Ugni Blanc *flb* LG 4, respectively. The LG 4 QTL was stable over half of the conditions. No repeated QTL was detected for other vegetative traits (BB, PHY, LMA and IL) that varied within environments.

#### Seed number, berry weight, number of berries and clusters

A new major QTL for the number of seeds per berry (NS) was found on Ugni Blanc *flb* LG 7 in all studied environments, where it explained 48 % to 76 % of the total variance (Table 3 and Fig. 2). This major QTL co-localized with the QTLs for berry weight at green lag phase (BWG) and at maturity (BWM), which explained 25-44 % and 17-42 % of total variance, respectively, in the different conditions investigated. Stable QTLs for the number of clusters (NC) and the number of berries per cluster (NB) were also localized in the same region, explaining 13-25 % and 18-24 % of total variation, respectively. Another repeated QTL for the number of berries per cluster (NB), explaining 13–18 % of total variance, was detected twice on LG 14 in Ugni Blanc *flb*.

#### Berry organic acid contents

Major and minor QTLs for malate and tartrate contents at green lag phase were identified in Ugni Blanc *flb* and Picovine. Five stable QTLs were discovered, for malate/total acid (MOG), malate/tartrate (MTG), and tartrate/total acid (TOG) ratios and for berry tartrate concentration (TaG) in Ugni Blanc *flb*, explaining from 12 % to 35 % of total variation*.* Four of them co-localized with the seed number and berry weight QTLs on LG 7. Another TaG QTL was identified on LG 4 in Ugni Blanc *flb*, but contrary to the LG 7 TaG QTL, it did not co-localize with QTLs for the dimensionless traits MTG, MOG or TOG. Only one minor repeated QTL for a berry acidity trait was detected twice in Picovine, at the top of LG 5, explaining 6 % to 12 % of the total berry acid concentration (ToAG) variance at green lag phase.

### Candidate genes

The size of integrated QTL confidence intervals (see Methods) varied from 3.1 to 14.0 Mb (Table 4) and harbored from 302 to 1201 genes per QTL. As a first approach, we screened these candidate genes taking into account their functional annotations (Additional file 8) and expression patterns (Additional file 9), which reduced by four to 28 times the number of most probable candidate genes per QTL (Table 4). The distribution of these selected candidate genes according to each main biological function is shown in Additional file 10.

**Table 4:** Integrated confidence interval limits for repeated QTLs and number of total and most probable positional candidate genes

					Number of candidate genes			Number of relevant candidate genes	
Traits	Chromosome	Start position (bp)	Stop position (bp)	Length (Mb)	CRIBI annotation	REFSEQ annotation	Total^b^	Involved in appropriate functions	And expressed in appropriate organs
LA	4	20322895	23912829	3.6	220	204	353	79	33
LA	19	1859933	4965830	3.1	231	185	377	41	25
ToAG	5	16515489	27520474	11.0	447	341	765	27	19
BWG	7	4916723	15195449	10.9^a^	400	320	617	122	65
BWM	7	6319558	14198046	7.9	261	227	400	102	62
MOG	7	5465273	16113558	10.6	383	300	654	40	16
MTG	7	5303765	16113558	10.8	399	316	686	40	16
NB	7	6177689	20219664	14.0	723	549	1201	86	52
NB	14	19704668	23504652	3.8	172	164	302	38	32
NC	7	8922964	15861847	6.9	200	126	306	23	11
NS	7	6461425	14101459	7.6	250	216	436	36	28
TaG	4	8840288	16951127	8.1	192	163	336	19	10
TaG	7	5096194	14716952	9.6	352	294	613	44	19
TOG	7	5303765	15673945	10.4	368	295	634	40	16

^a^10.3 Mb from chromosome 7 and 0.6 Mb from Unknown chromosome according to the genetic map

^b^Some genes are common between the two annotations

## Discussion

This QTL study, merging extensive phenotyping data (up to 43 traits, including five vegetative ones and 38 reproductive ones, assessed in nine environments) with a high-density genetic map obtained with the 18 K SNP Chip, led to identify 10 new stable QTLs. Some traits regarding berry acidity were mapped in *Vitis vinifera* for the first time and new genome regions were identified for these and other traits. QTL stability assessment was expanded towards an unprecedented temperature variation range (average T°max - T°min) thanks to the possibility to grow the microvine progeny in tightly controlled conditions, which is almost impossible with standard non-dwarf vines.

### Segregation extent and heritability of phenotyped traits in the population

The dwarf mapping population showed berry weight and composition variations consistent with those generally reported for grapevine. Indeed, berry weight of extreme individuals ranged from 0.2 g to 2.2 g at green lag phase, and from 0.5 g to 3.2 g at maturity stage. Similar variations were reported by Houel *et al.* [54] on a set of 165 *V. vinifera* wine varieties, including the ones used to generate the progeny: *cv*. Ugni Blanc and Pinot Meunier. Similar variation extent was also reported by Doligez *et al.* [25] in a segregating population from a cross between two other cultivars, Syrah and Grenache. In accordance with previous results on *V. vinifera* [55], the average total acid and potassium concentrations in fruits within the population were 509 and 53 mEq/kg.FW, respectively, at green lag phase. They decreased to respectively 197 and 87 mEq/kg.FW at berry maturity. The variation magnitude for total acid and potassium concentrations in ripe fruit observed between extreme individuals (3 to 5 fold) was the same as in another *V. vinifera* progeny (unpublished data).

These results indicate that, for reproductive traits, the Picovine 00C001V0008 x Ugni Blanc *flb* (*V. vinifera* L.) progeny behaved like other *V. vinifera* progenies. Interestingly, a correlation between glucose plus fructose and malate concentrations emerged at the green lag phase (Fig. 1b), namely before the onset of ripening, which was not documented before. Increased total sugar concentration is not an artifact due to the casual presence of ripe berries in green lag phase samples, since this would have resulted in a decrease in malate, conversely to what was actually observed. The level of sugars at the end of the first berry growth phase remains quite low and this illustrates that organic acids are by far the major osmoticum as compared to sugars, the opposite being true during the ripening phase (Additional file 1). Moreover, our results also suggest that malate, as a lower cost osmoticum, becomes even more favoured upon the impairment of the carbon balance, in different genotype x environment conditions.

In our study, some traits displayed lower broad-sense heritability than in previous studies, particularly acid or sugar-related traits at maturity. In previous studies, broad-sense heritability was most often above 0.5. At maturity, it was 0.61-0.94 for total sugar content [56, 57], 0.68-0.91 for malic acid and 0.47-0.75 for tartaric acid contents [56], 0.53-0.90 for total acids content [56, 58], 0.49-0.93 for berry weight [54, 58–62], 0.34 for seed number [59], 0.43 for number of berries per cluster [62], 0.55-0.94 for number of clusters [57, 62]. Broad-sense heritability was 0.96 for berry weight at *véraison* [54] and 0.67-0.82 for leaf area [48]. The temperature range explored in our study was very large thanks to the use of growth rooms (Additional file 11), and environmental variation may be inflated in our study compared to previous ones, especially to those reporting within-year heritabilities. This may partly explain the discrepancy between our estimates and the previous ones. Another possible explanation arises from the various ways maturity stage is assessed among studies (fixed *véraison*-maturity time-lag, seed color change, *etc*.; note that in many studies, maturity stage is not even defined). This may have biased genetic variance estimates in some studies. Last but not least, genetic variation and thus heritability strongly depends on the QTLs segregating in each cross, as suggested by the large range of estimates among studies for a given trait. In particular, genetic variation is expected to be larger in interspecific crosses than in pure *V. vinifera* ones.

### New QTLs for berry yield components

In addition to the number of clusters per axis, berry weight and number per cluster are key determinants of grapevine yield. QTLs for the number of seeds per berry (NS) and berry weight (BWM) in one or more years were already reported on linkage groups 2, 4, 8, 18 and 1, 5, 8, 11, 12, 13, 15, 17, 18, respectively [24–26, 63–66]. But this is the first time that major QTLs for NS, BWG and BWM are detected on LG 7 in grapevine. The parents of the present cross were related to wine cultivars from Northern and Western France (Pinot and Ugni Blanc), whereas the parents in previous *V. vinifera* QTL studies for these traits were wine cultivars from Southern France and Spain (Syrah and Grenache) or related to table cultivars (Big Perlon, Muscat, Sultanine, *etc*.) from Italy, Spain, Eastern Europe, *etc*. Therefore, since different selection histories have certainly produced various heterozygosity status among these parents, it is not surprising to find novel QTLs in the present study.

Moreover, QTLs for NS, BWG and BWM co-localized on LG 7 and showed decreasing variance, suggesting that a major locus might affect seed and berry cell numbers simultaneously during early development, or alternatively that expansion might indirectly be controlled by seeds through growth regulators control, later on in the development [67]. This result is consistent with the co-localization of seed trait QTLs with the major berry weight QTL on LG 18 in the seedless context [24, 25, 63–65], but contrasts with the lack of co-localization of any other seed trait QTLs with berry weight QTLs in any cross reported to date in grapevine. The consequences for use in breeding will therefore differ for this particular locus. The high correlation between BWG and BWM in this population is consistent with our previous finding on a sample of 254 varieties of *Vitis vinifera.* Indeed, the main determinants of the genetic variation for berry size were shown to be active before the green lag phase of berry growth [54].

Stable QTLs were also identified on LG 7 for the number of berries per cluster and the number of clusters per phytomer (NB, NC) and a repeated one was found on LG 14 for NB. Only the NB QTL on LG 7 co-localized with a similar one identified by Fanizza *et al.* [26] in one year only.

### Grape berry acidity QTLs

Grape berry acidity is known to be severely impacted by temperature during the growing season and should become a target of prime importance for breeding [68–70]. We showed here that malic acid may be strongly impacted by temperature during the green growth stage, and that the malate/tartrate ratio may strongly vary, depending on environmental conditions, while the total acid concentration is more stable (Additional file 1). Here, several stable QTLs regarding berry organic acid contents at green lag phase were identified for the first time in a pure intra-specific *V. vinifera* cross. Chen *et al.* [71] recently reported two-year repeated QTLs for malate and tartrate/malate ratio on LG 18 in a complex interspecific cross between several *Vitis* species. Two major tartrate concentration (TaG) QTLs were detected on Ugni blanc *flb* LGs 4 and 7, explaining each from 12 % to 35 % of total variance. They are the first stable significant tartrate QTLs reported in grapevine. A single-year phenotyping study previously led to the identification of putative only QTLs for berry pH and tartaric acid concentration in an interspecific cross [66]. According to our results, it will be possible to modify tartrate concentration in berries by breeding within *V. vinifera*, without resorting to interspecific crosses. This is a highly valuable result, since interspecific introgression schemes are more complex and introduce some undesired characteristics in wine taste, which are not widely accepted, interspecific hybrids even being often merely forbidden.

Tartrate synthesis occurs quite rapidly following fecundation. Then, its concentration decreases, due to dilution, while malate and sugars become the major osmoticum in green and ripe berries, respectively. Such a mechanism makes TaG dependent not only on tartrate synthesis, but also on berry expansion and malate synthesis. Dimensionless calculated traits such as the malate/tartrate ratio or the tartrate or malate relative contribution ratios (MTG, MOG or TOG) confirmed the LG 7 acidity QTL in all environmental conditions investigated. Puzzlingly, this was not the case for the LG 4 QTL, suggesting that these QTLs could act through the genetic control of intrinsically different events. In this respect, the co-localization of seed number, berry weight, and malate/tartrate QTLs on LG 7 may not be circumstantial. Its most parsimonious interpretation is that a single gene expressed during early berry development would affect seed number, which in turn would drive malate synthesis and cellular expansion, which is linked to increased malate/tartrate ratio [52]. Further experiments addressing cell number and the kinetics of malate and tartrate accumulation on extreme phenotypes are needed to confirm these hypotheses.

### QTLs for leaf area and other traits

In this study, two QTLs have been identified for leaf area (LA) on LGs 4 and 19. Two previous studies reported QTLs for leaf morphology and area in grapevine [48, 72] that did not co-localize with our repeated LA QTLs. However, one LA QTL identified only once (Additional file 7) co-localized with one QTL mentioned by Coupel-Ledru *et al.* [48] on LG 17. These discrepancies between studies highlight the polygenic determinism of berry weight, seed number and leaf area, with different genes or alleles segregating in different populations.

In this study, QTLs for PHY, IL, PIF, PFV, MaG, CiG, COG, CiM, MOM, TOM, COM, MTM, ToSG, KG, ASKG, GFM, ToSM and KM traits were found in one growing condition only (Additional file 7), suggesting frequent occurrence of genotype x environment interactions. For some other traits (BB, LMA, NBI, PBI, SW, MaM, TaM, ToAM, GuG, FuG, GFG, GuM, FuM, ASKM), no significant QTL was detected. For some of these traits, especially those with a low heritability, the parents of the cross might simply not be heterozygous for the main underlying genes. For the other traits, the reason might be the limited power for detecting small QTLs which results from the limited population size. Moreover, the berry weight QTL was detected in fewer environments at fruit maturity than at green lag phase. Furthermore, the QTL of berry tartrate content identified at green lag phase disappeared at maturity. This may reflect increased berry sampling errors due to the increase of berry heterogeneity during ripening or to inaccurate assessment of ripe stage, in the absence of precise kinetic measurements.

### Co-localization of QTLs and correlations

Nine berry or organic acid-related QTLs co-segregated on LG 7. Some of these traits were highly correlated, based on the Ward hierarchical classification. The negative correlation between number of berries (NB) and number of clusters (NC) likely results from plant physiological limitation, possibly insufficient carbon supply, to allow for fruit development and ripening. QTLs for NB and NC had small effects but also small heritability. QTLs for berry weight had large effects compared to their *H*^2^. Therefore, their co-localization on LG 7 alone could explain their observed correlation. Final berry weight is determined early during berry development and organic acids constitute the major osmoticum for vacuolar enlargement during the green growth stage, supporting a nine-fold increase of the berry cell volume between anthesis and the onset of ripening [73].

Finally, the lack of phenotypic correlation between traits showing QTLs co-localized on LG 7 might be explained by other QTLs, not detected in this study and not co-localized, but also by a lack of environmental correlation. Although leaf area (LA) and internode length (IL) were positively correlated (Spearman *ρ* = 0.71 over all environments, Fig. 1a) and heritability was slightly higher for IL than for LA, repeated QTLs were found only for LA and not for IL, suggesting that this newly reported correlation was mainly of environmental rather than of genetic origin.

### QTLs stable under different environments

In grapevine, two studies on the genetic determinism of adaptation to water stress allowed the identification of QTLs involved in the acclimation of scion transpiration induced by rootstock [47] and in the regulation of leaf water potential under soil drought partly due to reduced leaf transpiration [48]. Selection of allelic variation at these QTLs appears to be a promising way to select new cultivars to face climate change.

In our study, although the population showed a response of both vegetative and reproductive traits to thermal chart variations (growth rooms experiment), no repeated QTL could be evidenced for trait difference between the two temperature conditions (Additional file 7). Since response to temperature exhibited a large variability for each trait, the absence of QTLs for this response seemed to be rather due to low heritability (data not shown). Nevertheless, 10 QTLs stable under different environments mainly differing in terms of temperature have been found. By design, in all environments, the progeny was grown in 3 L pots with the same substrate and non-limiting irrigation. Moreover, in using growth rooms, our objective was to obtain differences only in temperature, since photoperiod, air vapour pressure and radiation level were regulated. These QTLs thus represent another very interesting genetic potential for the delivery of new cultivars with stable yield and quality under warmer climate conditions.

### Candidate genes

The integrated confidence intervals around repeated QTLs (from 3.1 to 14.0 Mb) were large, harbouring several hundred genes. Such interval sizes make the identification of candidate genes particularly tricky, insofar as gene annotation remains perfectible in grapevine. Low acidity phenotypes were recently attributed to mutations in an aluminium activated malate transporter in apple, and in an uncharacterized transporter in Cucurbits (MDP0000252114 [74]; XP_008463303 [75]), but no genes co-localizing with acidity QTLs in *Vitis* exhibited significant homologies with them (BLASTP, data not shown). Moreover, organ specific traits may be indirectly controlled by genes expressed elsewhere in the plant. Keeping these reserves in mind, as a first approach, we have screened candidates using the last annotation releases from both CRIBI and NCBI and selected a set of genes showing positive expression patterns in targeted organs, thus lowering down the candidate gene number to 10 to 65 per QTL. None of these genes had been previously identified in QTLs for fruit size [38, 39, 76–83] or fruit acidity [75, 84, 85] in fleshy fruit crops. One of the positional candidate genes from the short list obtained is a putative cytoplasmic Malic Dehydrogenase (MDH; VIT_207s0005g03350 from CRIBI annotation, LG7). This enzyme is involved in the conversion of malate into oxaloacetate together with other isoforms in mitochondria and plastids [86–89].

In any case, this study put forward a first list of candidate genes which should be confronted with data from association genetics or transcriptomic studies for validation. Considering the number of somatic variants available for grapevine [90], mutants affected for these traits, such as the fleshless berry mutant or the reiteration of reproductive meristems mutant [38, 39] may also be used for this purpose.

### The microvine: a valuable tool for QTL mapping

The Microvine or Dwarf and Rapid Cycling and Flowering (DRCF) mutant was recently proposed as a new model system for rapid forward and reverse genetics [51]. It is relevant for genetic studies on grapevine as it can be used as an annual crop, while presenting all characteristics of a perennial crop. It offers several advantages when compared to a non dwarf genotype: (1) a compact size, allowing the study of entire microvine populations under controlled environment, (2) an early flowering that occurs in the same year as sowing, instead of 4–6 years with the non DRCF genotypes, and (3) a continuous production of reproductive organs with sequential ripening allowing the study of all the development stages at the same time or at several times during the year. Such a sequential ripening along the shoot is known to occur in non-DRCF vines as well [91]. These characteristics are ideal to prospect the genetic and ecophysiological bases of grapevine adaptation to abiotic stresses, since microvine berry development exhibits the same pattern as regular vine [45, 92, 93]. Using microvine progenies and high throughput microarrays screening, Fernandez *et al.* [38] were able to map the fleshless berry locus and to identify a mutation in *VvPI* as the origin of the fleshless berry phenotype. Moreover, Dunlevy *et al.* [94] used a F2 progeny of a cross between a DRCF mutant, which does not produce 3-isobutyl-2-methoxypyrazine (IBMP), and the *V. vinifera* Cabernet Sauvignon cv., to identify the major locus responsible for accumulation of IBMP in grapes.

Microvine was used in the present study to decipher the genetic control of quantitative traits related to plant vegetative and reproductive development. The microvine population, obtained from a cross between a Picovine x Ugni Blanc *flb,* allowed the phenotyping of up to 43 traits under nine environmental conditions. However, to obtain a large microvine mapping population, the use of the Picovine as a female parent was required, because it is homozygous for the dwarf mutation (*Vvgai1*) and female loci [51]. The high homozygosity of the Picovine 00C001V0008 genome, resulted in only half a maternal genetic map, with an entire linkage group missing (LG 7). Thus, the identification of QTLs for this parent was not exhaustive.

### The grapevine 18 K SNP chip

The 18 K SNP chip allowed building both high-quality and high-density genetic maps. Indeed, the overall genotyping error rate was ≤ 0.0005 for each map, and only 167 out of the 18,071 SNPs present on the chip were discarded due to segregation distortion issues. In addition, reproducibility of control genotypes used for the chip creation was 100 %, when the DNA analysed was of good quality (A. Launay, personal communication). This was the case for all the samples in the present study. Such a very low error rate is an advantage of this high-throughput technique when compared to bar-coded multiplex sequencing [95] or Genotyping By Sequencing [96], which produce huge amounts of data but with a high rate of genotyping error.

The two high-density parental genetic maps contained 408 and 714 SNP markers with an average distance of 1.8 and 1.5 cM for Picovine and Ugni Blanc *flb*, respectively. The marker coverage of these genetic maps is higher than in most recent studies using AFLP, SSR and/or SNP markers in grapevine. The latest studies reported an average interval between adjacent markers from 1.9 to 7.3 cM for genetic maps with less than 300 markers per map [25, 34, 66, 82, 97–100]. The map of Vezzulli *et al.* [49] was based on 1,134 markers with an average spacing of 1.3 cM, but it resulted from the integration of maps from three different populations. Recently, Wang *et al.* [101] and Barba *et al.* [36], using next generation sequencing, reported parental maps of 759–1,121 SNP markers with inter-marker distances of 1.7-2.3 cM and a consensus map of 1,215 SNP markers distant of 1.6 cM on average, respectively. Recently, Chen *et al.* [71] also reported two parental maps with intervals ranging from 2.0 to 2.5 cM, by genotyping an interspecific *Vitis* hybrid with next-generation restriction site-associated DNA sequencing.

Here, the high average map density achieved was fully satisfying since maps were saturated with many co-segregating SNP markers, despite the low proportion of informative markers (6,000 out of 18 K) in the mapping population. In previous studies using high-throughput Illumina® SNP chip genotyping for QTL or association genetics in rice, alfalfa, maize and wheat [102–105], the proportion of polymorphic markers was larger, ranging from 52 % to 81 %. The 18 K grapevine SNP chip was composed of 13,561 SNPs (75 %) from 47 *Vitis vinifera* and 4,510 SNPs (25 %) from 13 other *Vitis* species and *Muscadinia rotundifolia* [106] while 96 % of the 6,000 SNPs polymorphic in the mapping population were from *V. vinifera*. This discrepancy partly explained the low proportion of SNPs that could be used for mapping in this population*.* Within the *Vitis* genus, species are clearly differentiated [107] and SNP transferability to *V. vinifera* is low [108]. In spite of the technical constraints for the design of specific probes [106], there were only two regions not covered with *V. vinifera* SNP markers on the chip*,* corresponding to the bottom of chromosome 9 (about 8.9 Mb) and to an inferior part of chromosome 3 (about 4.4 Mb) (A. Launay, personal communication). The technical constraints, together with the low polymorphism levels of non-*vinifera* SNP markers in this population could explain the few gaps observed in parental maps, their occurrence being further increased for Picovine due to its high homozygosity.

## Conclusions

Applying an abiotic stress on a whole population for genetic studies is particularly difficult for a perennial crop such as grapevine. Thanks to the reduced size of the microvine and its biological characteristics, we were able to grow a progeny of microvines under several environmental conditions, mainly differing in temperature. In this study, we identify some new QTLs for important developmental vegetative and reproductive traits that have limited interactions with environmental factors such as temperature. Therefore, these QTLs are a valuable first step towards finding useful genetic variation for maintaining vine yield and fruit quality under elevated temperatures.

## Methods

### Plant material and growth conditions

**Fig. 3:**
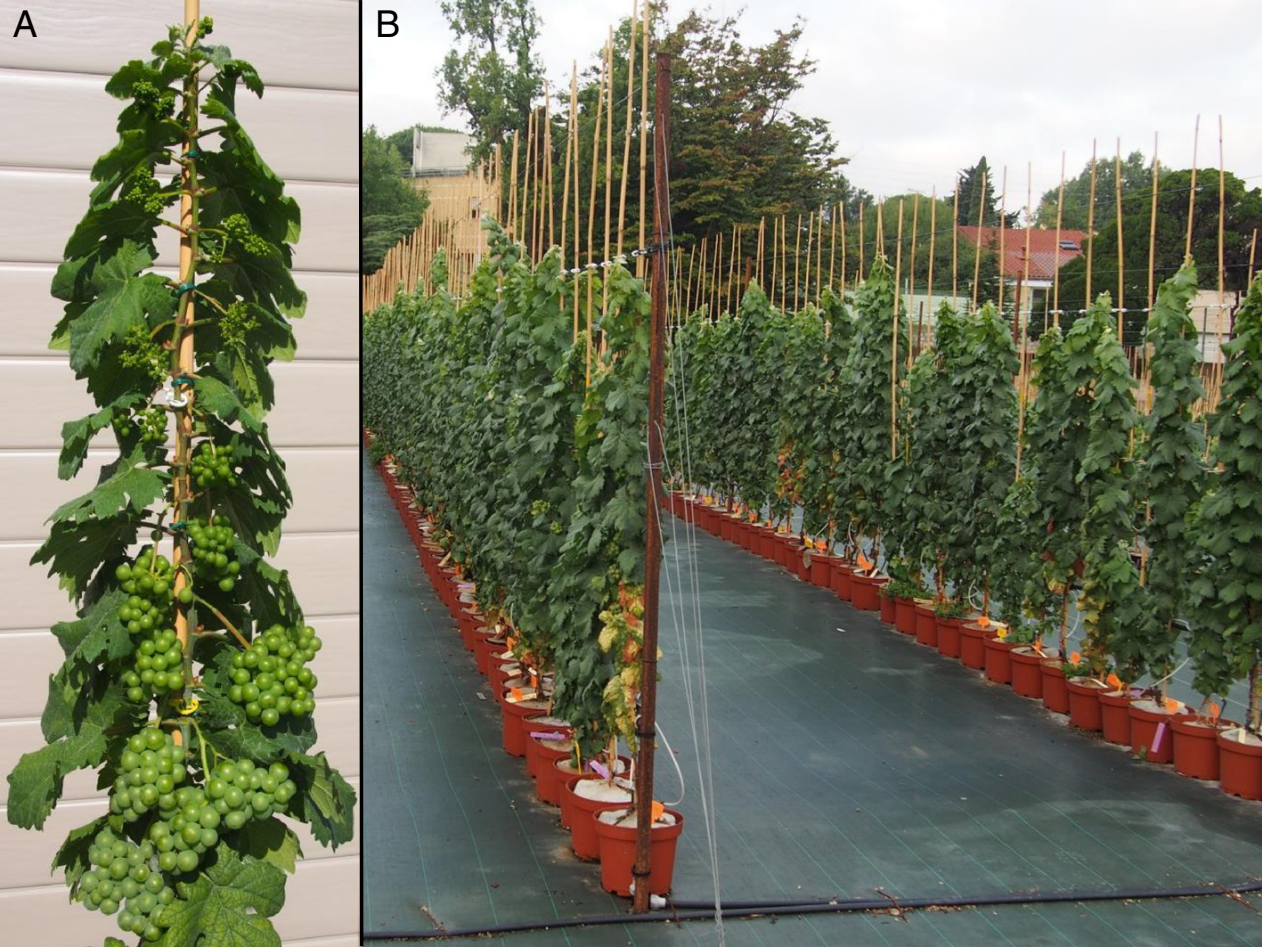
The microvine mapping population derived from the cross between Picovine and Ugni Blanc *flb*. (A) Microvine plant with continuous reproductive development along the proleptic axis. (B) The population grown outdoors in pots

The present study was performed at Montpellier SupAgro-INRA campus (France) on a pseudo-F1 microvine population from 2011 to 2014. The latter was obtained from a cross between the Picovine 00C001V0008 (*Vvgai1*/*Vvgai1*), which confers to the progeny Dwarf and Rapid Cycling and Flowering (DRCF) traits [51], and the grapevine Ugni Blanc fleshless berry mutant (*flb;* [52]). Only hermaphrodite individuals bearing wild type (non-fleshless) berries were retained, resulting in the selection of 129 microvine offspring in this progeny. In addition to the dwarf stature, an interesting biological property of the microvine is the continuous production of inflorescences along all the vegetative axes straight from the first year of development (Fig. 3a), with sequential ripening along the shoot [45, 92]. Several copies of each individual of this progeny were established in 3 L pots filled with Neuhauss Humin-substrate N2 (Klasmann-Deilmann, Bourgoin Jallieu, France). Three year-old plants were used for a better balance between root and above ground organ developments. Plants were spur-pruned to 2–4 buds. Then, a single proleptic axis was kept per plant close after budburst, in order to synchronize development between plants (Fig. 3a). Sylleptic axes were removed as soon as they appeared to reduce crop load. At budburst, 15 g of Osmocote exact standard fertilizer (Everris, Limas, France) were added. Non-limiting irrigation was supplied during the whole plant cycle (Fig. 3b). One copy of the population was grown in a greenhouse and two copies were grown outdoors in two complete blocks. In order to identify stable QTLs across more varied thermal growth conditions, two copies were also grown in growth rooms under controlled environments, during one month. Temperature treatments were 20°/15 °C and 30°/25 °C (day/night) for “cool” and “hot” treatment, respectively. Each treatment was applied to a single copy of the population. A 14-h photoperiod was imposed. In the growth rooms, mean Vapour Pressure Deficit (VPD) was maintained between 0.7 and 1.8 kPa during photoperiod and average daily Photosynthetic Active Radiation (PAR) per day was around 20–25 mol.m^−2^. The different climatic conditions during plant growth are summarized for all environments in Additional file 11.

### Phenotypic variables

Forty-three traits (five vegetative traits and 38 reproductive traits; Table 1) were either directly measured or inferred from direct measurements, on one copy of the population in the greenhouse in 2011, on one copy (2011) and two copies (2012, 2013 and 2014) outdoors, and on two copies in growth rooms in 2013 and 2014 (Table 1).

#### Vegetative traits

Budburst time (stage EL4; [109]) was determined from cumulated growing degree-day (GDD) after March 15^th^. GDD was calculated as the difference between the average of the daily temperatures and the base temperature (T_base_ = 10 °C; [110]). The number of unfolded leaves per vine was counted twice a week for two months in the greenhouse and outdoors, and during the whole experiment (one month) in growth rooms. The leaf emergence rate was calculated from linear regression between the cumulated GDDs after budburst and the number of leaves. The phyllochron (PHY), or GDD required between the emergence of two successive leaves, was the reverse of the leaf emergence rate. Leaf area (LA) was calculated from leaf main vein length measurements. Specific allometric relationships between the above variables were parameterized for each genotype from measurements on seven leaves of constrasted plastochron index, using ImageJ version 1.43 software (National Institutes of Health, Bethesda, Maryland, USA). Six leaf disks of 1 cm diameter were sampled on each plant and dried at 70 °C for 72 h to determine leaf mass per area (LMA). The internode length (IL) was calculated at the end of the experiments as the whole proleptic axis height divided by the number of phytomers in the greenhouse and outdoors, or just considering unfolded phytomers during temperature treatments in growth rooms.

#### Reproductive traits

The number of pre-formed basal inflorescences (*i.e.* inflorescences differentiated within winter buds) per plant (NBI) and the position of the first pre-formed inflorescence (PBI) on the main proleptic axis were noted. The pre-formed basal inflorescences could be distinguished from the neo-formed ones, because they were larger, with more branching and more flowers and located at ranks 3 to 6 on the proleptic axis. Basal inflorescences were removed after flowering to avoid a competition with neo-formed inflorescences. The period from inflorescence appearance (stage 51 according to BBCH international scale; [111]) to 50 % flowering (stage 65) (PIF) and from 50 % flowering to 50 % *véraison* (stage 85) (PFV) were observed on three neo-formed clusters per plant. All the berries of two clusters were sampled at two developmental stages at the herbaceous plateau and 40 days after the onset of ripening, thereafter called ‘green lag phase’ and ‘maturity stage’, respectively. The continuous reproductive development and sequential ripening along the main axis of microvine plants allowed an accurate assessment of the onset of ripening, characterized by berry softening. Berries just prior to this stage, on the former younger phytomer, were considered to be at the ‘green lag phase’. For the ‘maturity stage’, inflorescences were tagged at the onset of ripening and sampled 40 days later. Two inflorescences per plant were tagged in the greenhouse and outdoors, and only one inflorescence per plant was tagged in growth rooms. At green lag phase and maturity stages, the berry fresh weight of seeded berries was recorded (BWG, BWM). At maturity, the total number of berries per cluster was counted, including seeded and seedless berries. Number of seeds (NS) and seed fresh weight (SW) were determined in seeded berries only. The number of clusters along ten successive phytomers (NC) was also recorded.

#### Berry biochemistry

Berries were randomly sampled at green lag phase and maturity stage. Depending on cluster size, 15 to 20 berries were crushed and diluted 5-fold in deionized water prior to freezing at −20 °C. For organic acids, glucose and fructose analyses, samples were thawed at room temperature and subsequently heated at 60 °C for 30 min. After return to room temperature, samples were homogenized and an aliquot was diluted 10 to 20 folds in 4.375 μM acetic acid (internal standard). To avoid potassium bi-tartrate precipitation and to reduce the area of the injection peak, 1 mL sample was mixed with 0.18 g of Sigma Amberlite® IR-120 Plus (sodium form), and agitated on a rotary shaker for at least ten hours before centrifugation at 13,000 rpm for 10 min. The supernatant was transferred to High Performance Liquid Chromatography (HPLC) vials before injection on an Aminex HPX®87H column eluted in isocratic conditions (0.5 mL.min^−1^, 60 °C, 0.5 g.L^-1^ of H_2_SO_4_) [112]. Organic acids were detected at 210 nm with a Waters 2487 dual absorbance detector® (Waters Corporation, Massachusetts, United States). A refractive index detector Kontron 475® (*Kontron* Instruments, Switzerland) was used to determine fructose and glucose concentrations. Concentrations were calculated according to Eyegghe-Bickong *et al.* [113] for deconvolution of fructose and malic acid, after checking the validity of this procedure on tartaric acid, malic acid, glucose and fructose standards, either in pure or mixed solutions. Several ratios between the biochemical components were also calculated (Table 1).

### Phenotypic data analyses

Phenotypic data were analysed using the R software version 2.15.0 [114]. Data were clustered using the Ward method as described in Houel *et al.* [54], in order to assess correlations between all traits for each growing condition. Normality of the distribution was tested for each trait, using the Shapiro-Wilk test [115]. When data distribution deviated from normality, a Box-Cox transformation [116] was applied to unskew the distribution. When trait data were available for two copies in a given environment, the full and sub-mixed linear models were adjusted using the lme4 package [117]. Then, the best-fit model was selected using the Bayesian Information Criterion (BIC). The full model was *Y*_ij_ = *μ* + *G*_i_ + *c*_j_ + *E*_ij_, where *Y*_ij_ was the phenotypic trait for copy *j* of genotype *i*, *μ* the general mean, *G*_i_ the random effect of genotype *i*, *c*_j_ the fixed effect of copy *j* and *E*_ij_ the random residual term. The best linear unbiased predictors (BLUPs) of genetic values were extracted for QTL detection when there were two copies. The genotype and residual variance estimates (*σ*^2^_G_ and *σ*^2^_E_, respectively) were used to estimate broad sense heritability (*H*^2^) of the inter-environment genotypic mean as *σ*^2^_G_/(*σ*^2^_G_ + *σ*^2^_E_), allowing for the possible addition of a fixed environment effect to the model. The assumption of normality of residual and BLUP distributions was checked through quantile-quantile plots comparing the observed distributions to a theoretical normal distribution.

### DNA extraction, SNP marker genotyping and marker selection

Deoxyribonucleic Acid (DNA) was extracted from 1 g of young leaves (with main rib less than 2 cm long) using DNeasy Plant Maxi Kit (Qiagen, Germany) following the manufacturer’s instructions. The concentration and quality of the DNA were checked using the Agilent® 2100 bioanalyzer system (Agilent, Santa Clara, CA, United States). The population was genotyped using the Illumina® 18 K SNP Infinium chip (18,071 SNP markers; [106]). Results were visualized and manually edited when necessary using the Illumina® Genome Studio software version 2011.1 [118]. The SNP markers that were monomorphic (55 % of the total markers), multilocus or with an ambiguous pattern (8 %), highly distorted or with a minor allele frequency < 10 % (1 %), were discarded. The remaining 6,000 SNP markers passing these filters were used to build the genetic maps, out of which 2,727 and 4,284 were heterozygous in Picovine and Ugni Blanc *flb*, respectively.

### Linkage map construction

For each parent, a framework linkage map of reliable order was constructed using CarthaGene version 1.0 [119], based on the most informative SNPs among the 6,000 usable ones. A LOD threshold of 4 and a distance threshold of 30 cM were used to identify linkage groups (LG). The grouping was also adjusted using the knowledge about physical genome map. The most likely marker order within each LG was determined using the stepwise marker insertion command “buildfw” (with 2, 0.2 and 1 for the Keep threshold, Add threshold and Mrktest arguments, respectively). This procedure yields a framework map by automatically selecting a subset of markers to ensure a reliable order. This order was then optimized using a taboo technique (“greedy” command with 3, 1, 1 and 15 for NbLoop, Fuel, TabooMin and TabooMax arguments, respectively). Finally, all possible permutations within a sliding window were applied to the best map obtained (“flips” command with 5, 2 and 1 for Size, LOD-threshold and Iterative arguments, respectively), to detect any better local order. The order and quality of the two genetic maps were then checked using the R package qtl [120], following the tutorial’s instructions [121]. The overall genotyping error rate was estimated within the 0.0005-0.05 range, the “checkalleles” function was used to detect markers with erroneous linkage phases and the “droponemarker” function to spot suspicious markers.

### QTL detection

QTL detection was performed in each parental map on the genotypic BLUPs when available for two copies or directly on transformed data, using the R qtl package. Multiple QTL regression was carried out with the "stepwiseqtl" function, as described by Huang *et al.* [32]. This approach is based on forward/backward selection to compare several multiple-QTL models with main effect QTLs and possible pairwise QTLxQTL interactions. To select the QTL model, specific penalties were applied to the LOD score according to the number of main effects and interaction terms. For each trait, these penalties were derived from 1000 permutations with a two-dimensional scan and a genome-wide error rate of 0.05. Genome scan was performed with a 1 cM step. LOD-1 QTL location confidence intervals were derived with the “lodint” function.

### Candidate genes for QTLs

When necessary, the confidence interval was first reduced to ±3 cM around the LOD peak of each QTL in each environment, in order to focus on the most probable location of the causative polymorphism [122]. Then, when the confidence intervals of a QTL in different growing conditions overlapped, the candidate genes were searched within the most extreme limits of the corresponding set of reduced overlapping intervals, thereafter referred to as the integrated interval. The physical coordinates of integrated interval limits on the latest version of the PN40024 reference genome sequence (assembly version 12X.2; [123]) were deduced from local recombination rate between flanking SNP markers with known physical coordinates [106]. Two public annotations of the genome were considered in order to maximize the chances to identify candidate genes: the latest CRIBI version 2 [124, 125] and the classical REFSEQ version 1 from NCBI [126], that both refer to the 12X.0 genome sequence. The gene coordinates in the CRIBI and the NCBI General Feature Format (GFF) files were corrected to take into account (1) scaffold rearrangements between PN40024 12X.0 and 12X.2 versions and (2) the insertion of 500 n between scaffolds in the CRIBI annotation that is absent in the NCBI one. All the coordinates given in the present paper refer to the PN40024 reference genome sequence assembly version 12X.2. As a first approach, based on this exhaustive list of positional genes, we performed a two-step selection to reduce the number of candidates per QTL. A list of the biological functions most probably associated with the identified QTL traits was established based on our own expertise and literature data [4, 127, 128] (Additional file 8). In this respect, the genes were selected according to the Gene Ontology available in the GFF files from both annotations. Lastly, the expression pattern of candidate genes in different organs and developmental stages of grapevine was retrieved from Fasoli *et al.* [129] in order to screen genes expressed in the organs linked to the traits for which QTLs were found.

## Additional files

Additional file 1: Figure S1. Phenotypic data distribution for the 43 traits under the different growing conditions. When the best model to estimate the BLUPs of genetic values of trait did not include a copy effect, the mean of the trait (M) was shown. Otherwise, the distributions of the two separate copies were shown (copy 1, copy 2). (PDF 244 kb) Additional file 2: Figure S2. Hierarchical classification of 43 traits under each growing condition. When two copies of the same traits were measured under the same environment, the mean value of the two copies was used in order to simplify the tree. Different colours represent trait categories for which a repeated QTL was identified. (PDF 616 kb) Additional file 3: Table S1. Correlation (Spearman coefficient) between environments. Suffixes G, F.1, F.2, cool and hot stand for greenhouse, first replicate, second replicate, cool and hot treatments during experiments in growth rooms, respectively. (XLSX 19 kb) Additional file 4: Figure S3 Framework parental genetic maps of Picovine and Ugni Blanc *flb* built with SNP markers from the 18 K SNP Infinium chip. (A) The Ugni Blanc *flb* genetic map. (B) The Picovine genetic map. (PDF 368 kb) Additional file 5: Table S2. Genome regions not covered by genetic maps (gaps > 5 cM within linkage groups, or > 1.6 Mb at the ends of linkage groups). (XLSX 13 kb) Additional file 6: Figure S4. SNP positions on genetic maps as a function of their physical position on the reference genome version 12X.2. The maternal parent is the Picovine (blue circles) and the paternal parent is the Ugni Blanc *flb* (pink circles). (PDF 321 kb) Additional file 7: Table S3. All significant quantitative trait loci (QTL) detected. (XLSX 20 kb) Additional file 8: Table S4. List of the biological functions most probably related to the mapped QTLs. (XLSX 10 kb) Additional file 9: Table S5. Physical localization and expression in different organs of the functional candidate genes potentially involved in the repeated QTLs identified. (XLSX 90 kb) Additional file 10: Figure S5. Distribution of the number of candidate genes expressed in appropriate organs, according to the main biological functions related to the repeated QTLs identified. (PDF 187 kb) Additional file 11: Table S6. Summary of the climatic data for the different experiments. (XLSX 15 kb)

## Abbreviations

ASKG: Total acids, sugars and potassium at green lag phase
ASKM: Total acids, sugars and potassium at maturity
BB: Budburst time
BIC: Bayesian Information Criterion
BLUP: Best Linear Unbiased Predictor
BWG: Berry weight at green lag phase
BWM: Berry weight at maturity
CiG: Citrate at green lag phase
CiM: Citrate at maturity
COG: Citrate/total acids ratio at green lag phase
COM: Citrate/total acids ratio at maturity
DNA: Deoxyribonucleic Acid
DRCF: Dwarfism, Rapid and Continuously Fruiting phenotype
FuG: Fructose at green lag phase
FuM: Fructose at maturity
GDD: Growing Degree Day
GFG: Glucose/fructose ratio at green lag phase
GFM: Glucose/fructose ratio at maturity
GFF: General Feature Format
GuG: Glucose at green lag phase
GuM: Glucose at maturity
HPLC: High Performance Liquid Chromatography
IL: Internode length
KG: Potassium at green lag phase
KM: Potassium at maturity
LG: Linkage Group
LA: Leaf area
LMA: Leaf mass per area
MaG: Malate at green lag phase
MaM: Malate at maturity
MOG: Malate/total acids ratio at green lag phase
MOM: Malate/total acids ratio at maturity
MTG: Malate/tartrate ratio at green lag phase
MTM: Malate/tartrate ratio at maturity
NB: Number of berries per cluster at maturity
NBI: Number of pre-formed inflorescences in winter buds per plant
NC: Number of clusters per ten phytomers at maturity
NS: Number of seeds per berry at maturity
PAR: Photosynthetic Active Radiation
PBI: Position of first pre-formed inflorescence
PHY: Phyllochron
PFV: Period from 50 % flowering to 50 % *véraison*
PIF: Period from inflorescence appearance to 50 % flowering
QTL: Quantitative Trait Locus
SNP: Single Nucleotide Polymorphism
SW: Seed weight at maturity
TaG: Tartrate at green lag phase
TaM: Tartrate at maturity
ToAG: Total acids at green lag phase
ToAM: Total acids at maturity
TOG: Tatrate/total acids ratio at green lag phase
TOM: Tatrate/total acids ratio at maturity
ToSG: Total sugars at green lag phase
ToSM: Total sugars at maturity
VPD: Vapour Pressure Deficit
