# Supplementary material for: Identification of stable QTLs for vegetative and reproductive traits in the microvine (Vitis vinifera L.) using the 18 K Infinium chip
Source: BMC Plant Biol. 2015 Aug 19;15:205. doi: 10.1186/s12870-015-0588-0 (PMC4539925; doi:10.1186/s12870-015-0588-0)
Supplement: Additional file 1: Figure S1. — Phenotypic data distribution for the 43 traits under the different growing conditions. When the best model to estimate the BLUPs of genetic values of trait did not include a copy effect, the mean of the trait (M) was shown. Otherwise, the distributions of the two separate copies were shown (copy 1, copy 2). (PDF 244 kb) [file 12870_2015_588_MOESM1_ESM.pdf]

Figure S1. Phenotypic data distribution for the 43 traits under different growth conditions. When the best model to estimate the BLUPs of genetic values of trait did not included a copy effect, the means of the trait (M) was shown. Otherwise, the distributions of the two separate copies were shown (copy1, copy2).

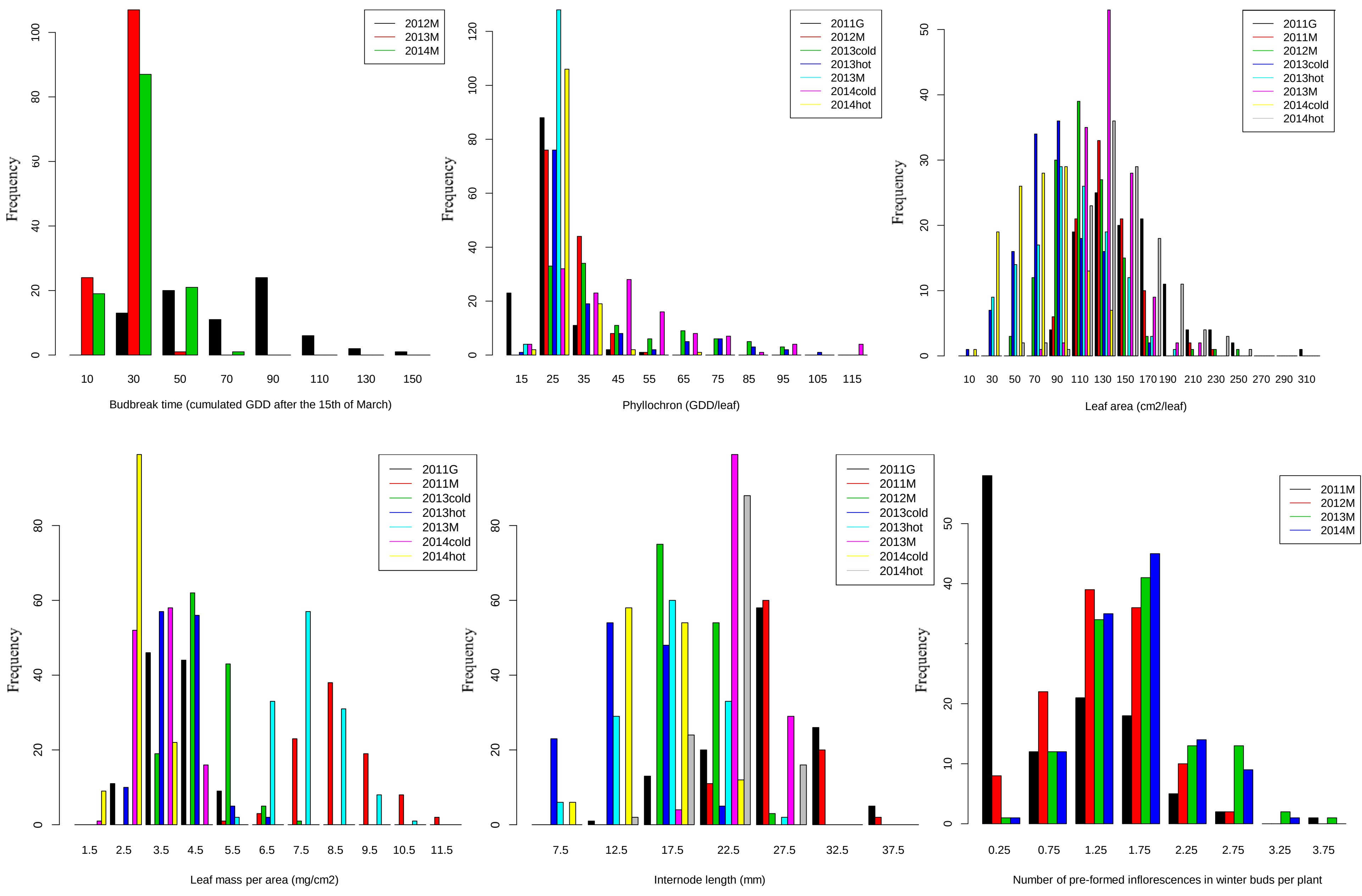

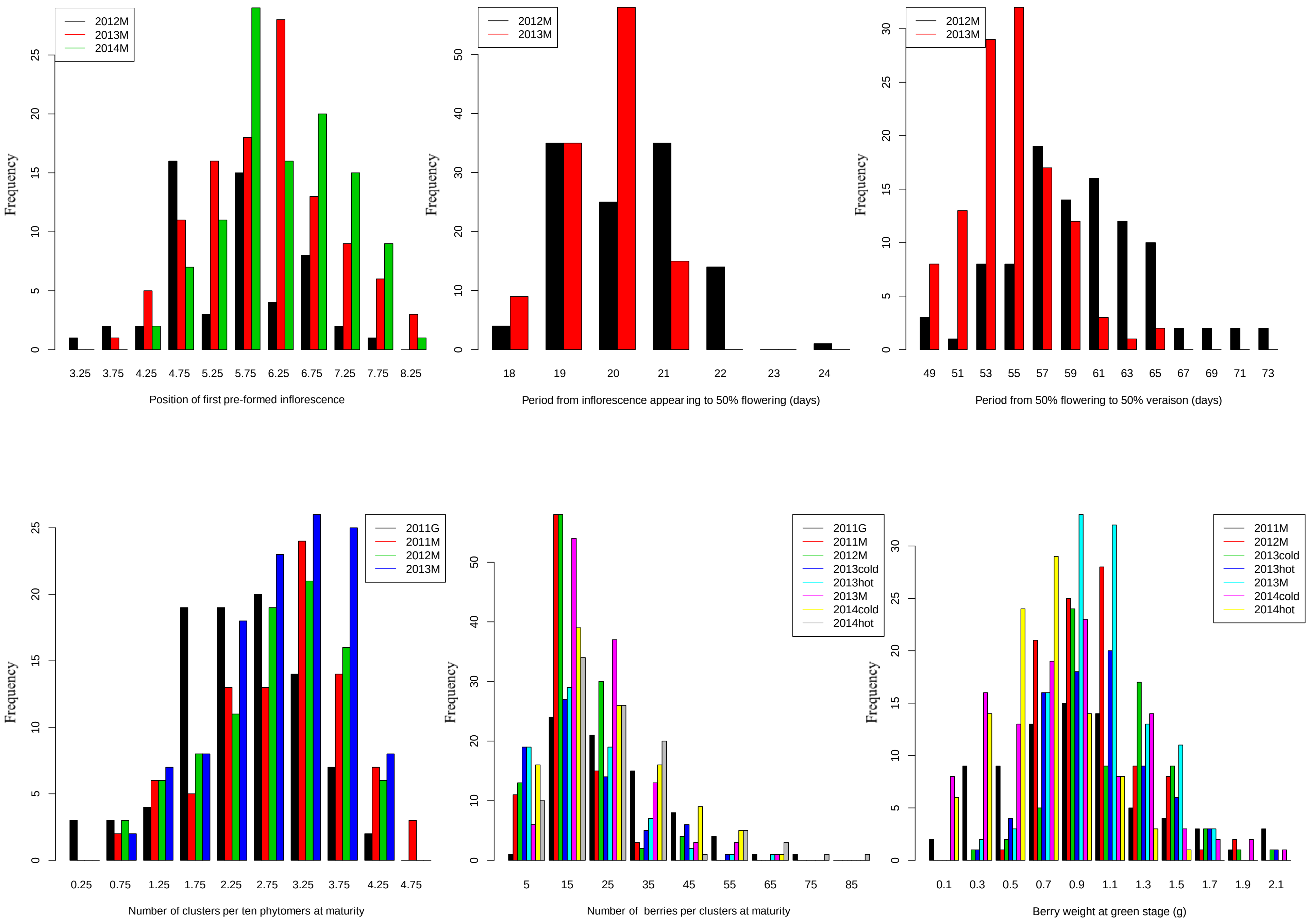
2012M
2013M
2014M
Frequency
3.25 3.75 4.25 4.75 5.25 5.75 6.25 6.75 7.25 7.75 8.25
Position of first pre-formed inflorescence
2012M
2013M
Frequency
18 19 20 21 22 23 24
Period from inflorescence appear ing to 50% flowering (days)
2012M
2013M
Frequency
49 51 53 55 57 59 61 63 65 67 69 71 73
Period from 50% flowering to 50% veraison (days)
2011G
2011M
2012M
2013M
Frequency
0.25 0.75 1.25 1.75 2.25 2.75 3.25 3.75 4.25 4.75
Number of clusters per ten phytomers at maturity
2011G
2011M
2012M
2013cold
2013hot
2013M
2014cold
2014hot
Frequency
5 15 25 35 45 55 65 75 85
Number of berries per clusters at maturity
2011M
2012M
2013cold
2013hot
2013M
2014cold
2014hot
Frequency
0.1 0.3 0.5 0.7 0.9 1.1 1.3 1.5 1.7 1.9 2.1
Berry weight at green stage (g)

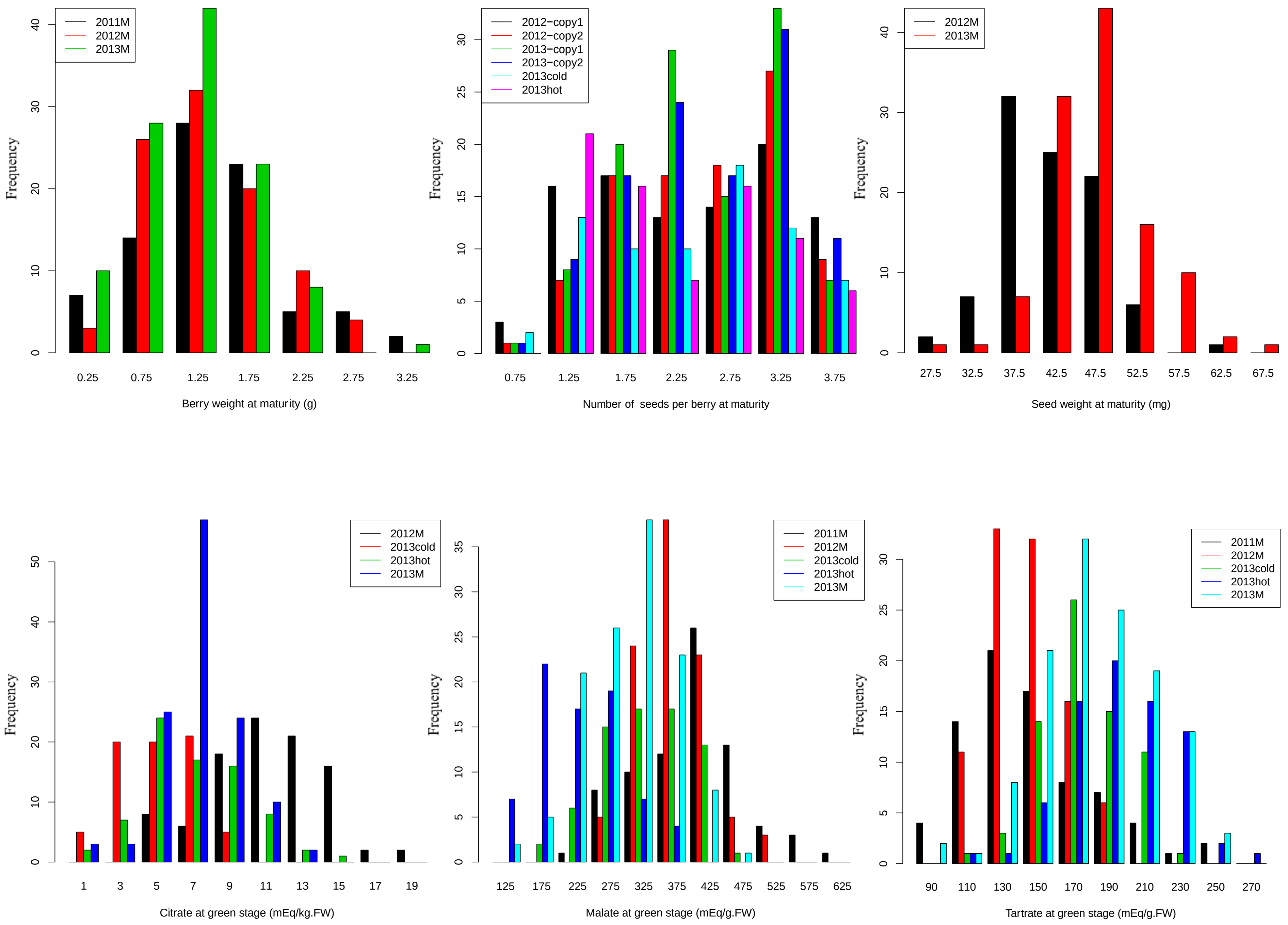
2011M
2012M
2013M
Frequency
Berry weight at maturity (g)
2012−copy1
2012−copy2
2013−copy1
2013−copy2
2013cold
2013hot
Number of seeds per berry at maturity
2012M
2013M
Seed weight at maturity (mg)
2012M
2013cold
2013hot
2013M
Citrate at green stage (mEq/kg.FW)
2011M
2012M
2013cold
2013hot
2013M
Malate at green stage (mEq/g.FW)
Tartrate at green stage (mEq/g.FW)

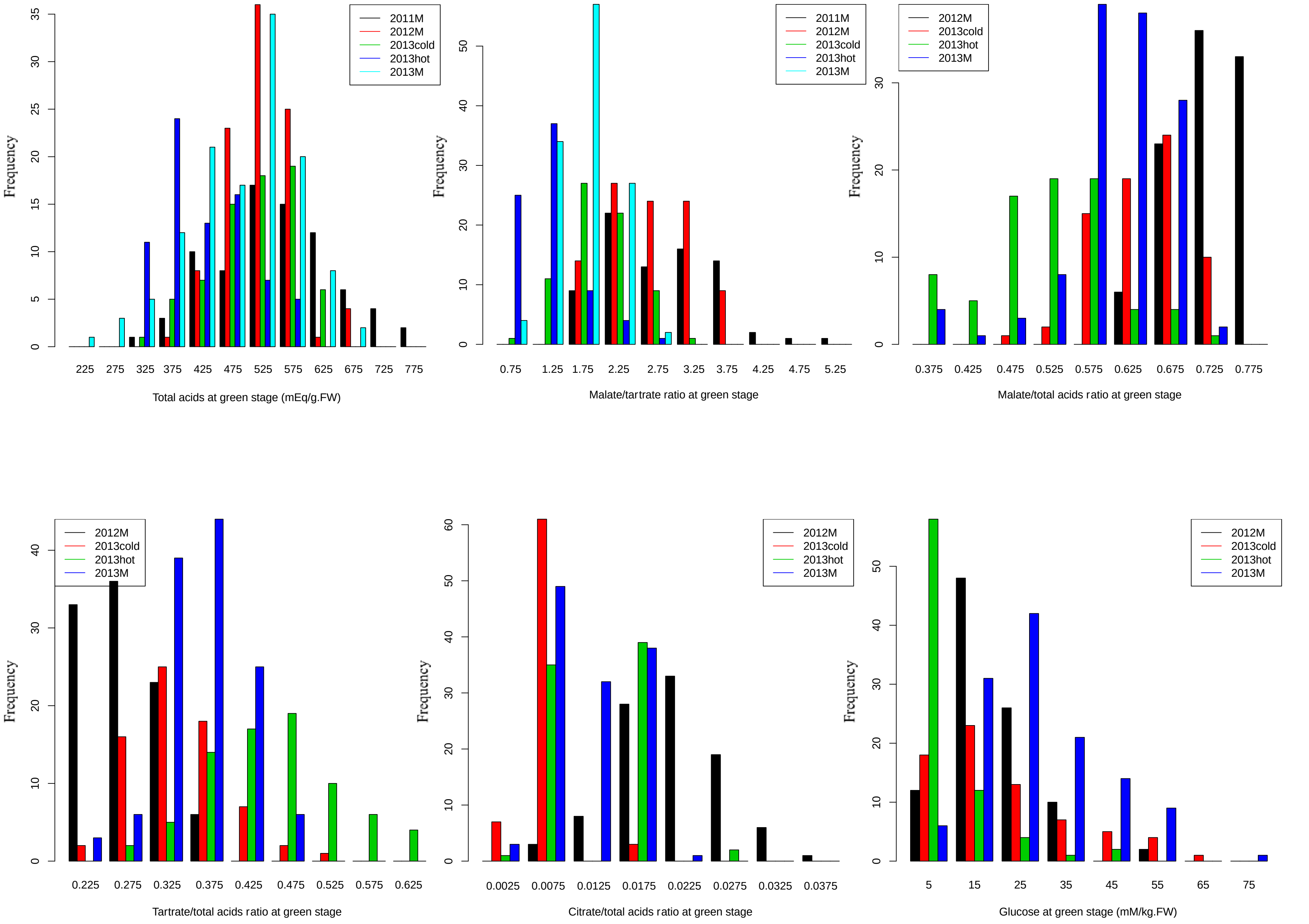

Frequency
2011M
2012M
2013cold
2013hot
2013M
225 275 325 375 425 475 525 575 625 675 725 775
Total acids at green stage (mEq/g.FW)
Frequency
2011M
2012M
2013cold
2013hot
2013M
0.75 1.25 1.75 2.25 2.75 3.25 3.75 4.25 4.75 5.25
Malate/tartrate ratio at green stage
Frequency
2012M
2013cold
2013hot
2013M
0.375 0.425 0.475 0.525 0.575 0.625 0.675 0.725 0.775
Malate/total acids ratio at green stage
Frequency
2012M
2013cold
2013hot
2013M
0.225 0.275 0.325 0.375 0.425 0.475 0.525 0.575 0.625
Tartrate/total acids ratio at green stage
Frequency
2012M
2013cold
2013hot
2013M
0.0025 0.0075 0.0125 0.0175 0.0225 0.0275 0.0325 0.0375
Citrate/total acids ratio at green stage
Frequency
2012M
2013cold
2013hot
2013M
5 15 25 35 45 55 65 75
Glucose at green stage (mM/kg.FW)

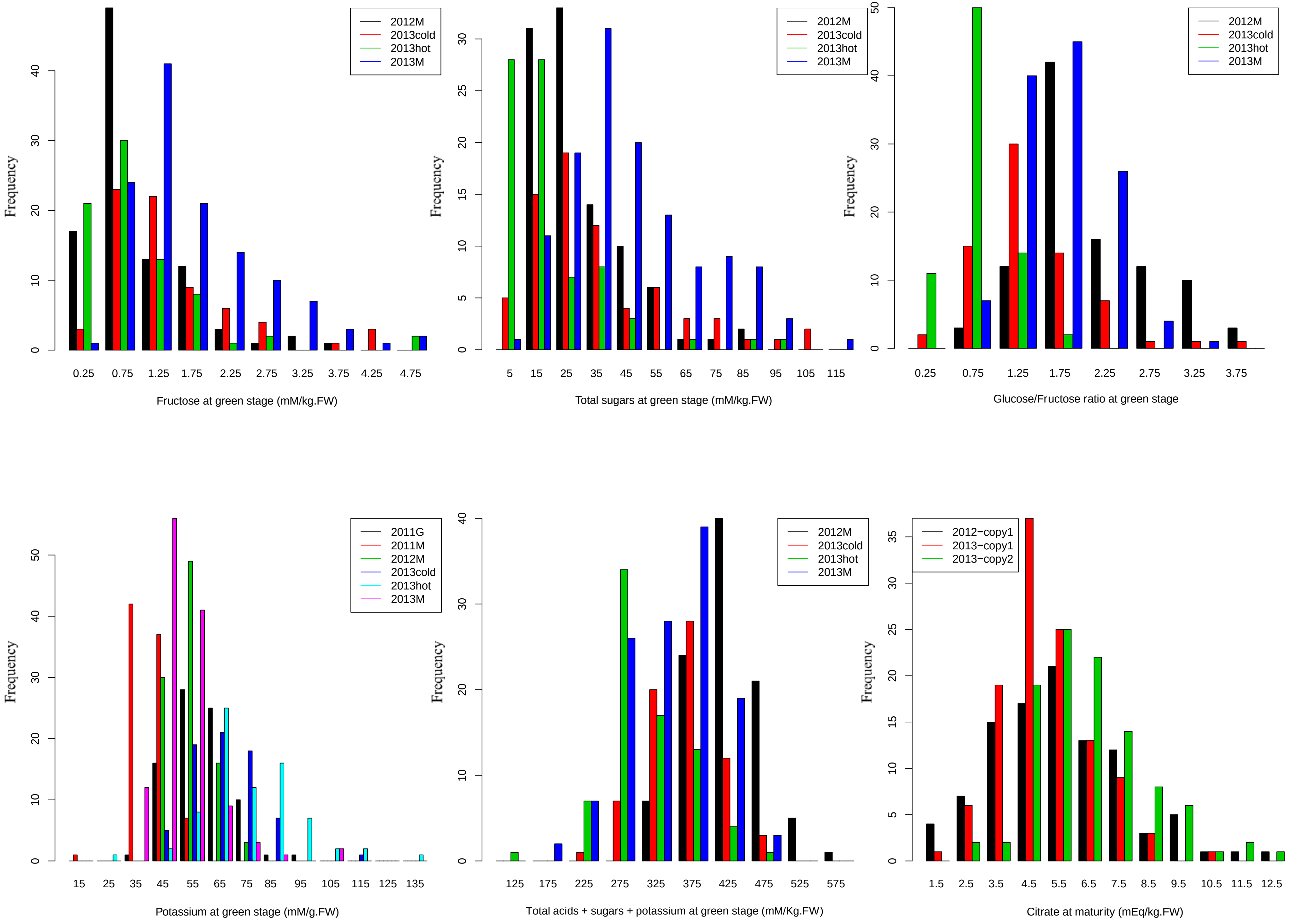
Frequency
2012M
2013cold
2013hot
2013M
Fructose at green stage (mM/kg.FW)
Total sugars at green stage (mM/kg.FW)
Glucose/Fructose ratio at green stage
2011G
2011M
Potassium at green stage (mM/g.FW)
Total acids + sugars + potassium at green stage (mM/Kg.FW)
2012−copy1
2013−copy1
2013−copy2
Citrate at maturity (mEq/kg.FW)

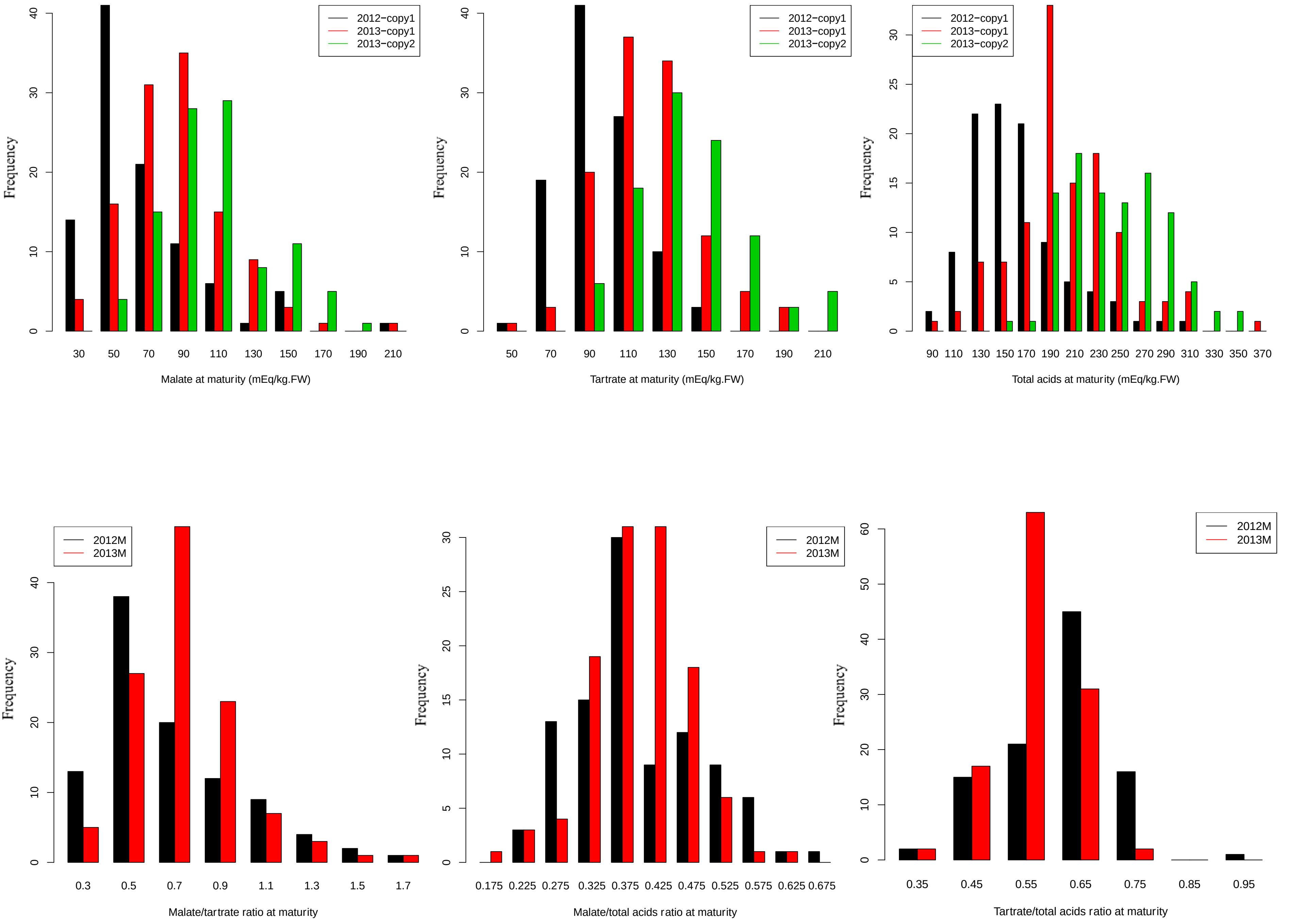
2012−copy1
2013−copy1
2013−copy2
Frequency
Malate at maturity (mEq/kg.FW)
Tartrate at maturity (mEq/kg.FW)
Total acids at maturity (mEq/kg.FW)
2012M
2013M
Malate/tartrate ratio at maturity
Malate/total acids ratio at maturity
Tartrate/total acids ratio at maturity

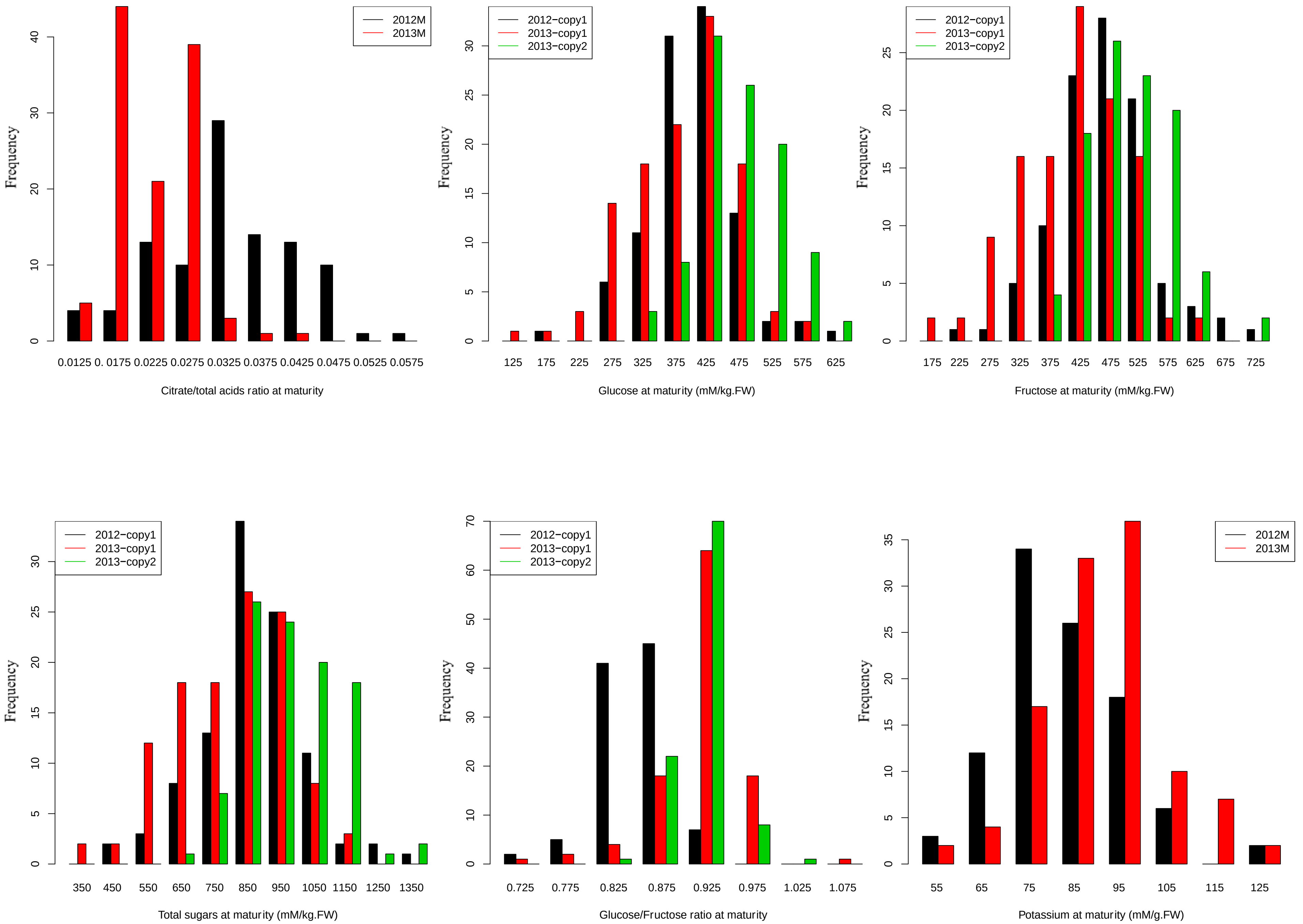

2012M
2013M
Frequency
0 10 20 30 40
0.0125 0. 0175 0.0225 0.0275 0.0325 0.0375 0.0425 0.0475 0.0525 0.0575
Citrate/total acids ratio at maturity
2012−copy1
2013−copy1
2013−copy2
Frequency
0 5 10 15 20 25 30
125 175 225 275 325 375 425 475 525 575 625
Glucose at maturity (mM/kg.FW)
2012−copy1
2013−copy1
2013−copy2
Frequency
0 5 10 15 20 25
175 225 275 325 375 425 475 525 575 625 675 725
Fructose at maturity (mM/kg.FW)
2012−copy1
2013−copy1
2013−copy2
Frequency
0 5 10 15 20 25 30
350 450 550 650 750 850 950 1050 1150 1250 1350
Total sugars at maturity (mM/kg.FW)
2012−copy1
2013−copy1
2013−copy2
Frequency
0 10 20 30 40 50 60 70
0.725 0.775 0.825 0.875 0.925 0.975 1.025 1.075
Glucose/Fructose ratio at maturity
2012M
2013M
Frequency
0 5 10 15 20 25 30 35
55 65 75 85 95 105 115 125
Potassium at maturity (mM/g.FW)

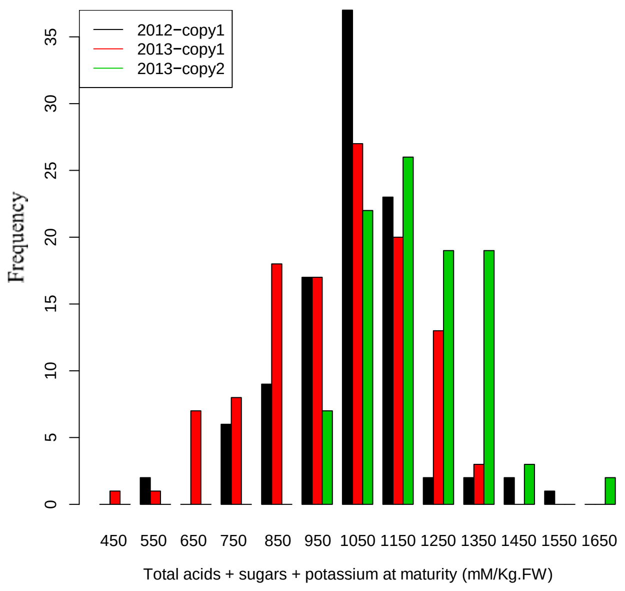
2012−copy1
2013−copy1
2013−copy2
Frequency
0
5
10
15
20
25
30
35
450
550
650
750
850
950
1050
1150
1250
1350
1450
1550
1650
Total acids + sugars + potassium at maturity (mM/Kg.FW)
