# Supplementary material for: Identification of stable QTLs for vegetative and reproductive traits in the microvine (Vitis vinifera L.) using the 18 K Infinium chip
Source: BMC Plant Biol. 2015 Aug 19;15:205. doi: 10.1186/s12870-015-0588-0 (PMC4539925; doi:10.1186/s12870-015-0588-0)

Figure S2. Hierarchical classification of 43 traits under each growing condition.
When two copies of the same traits were measured under the same environment, the mean value of the two copies was used in order to simplify the tree. Different colours represent trait categories for which a stable QTL was identified.

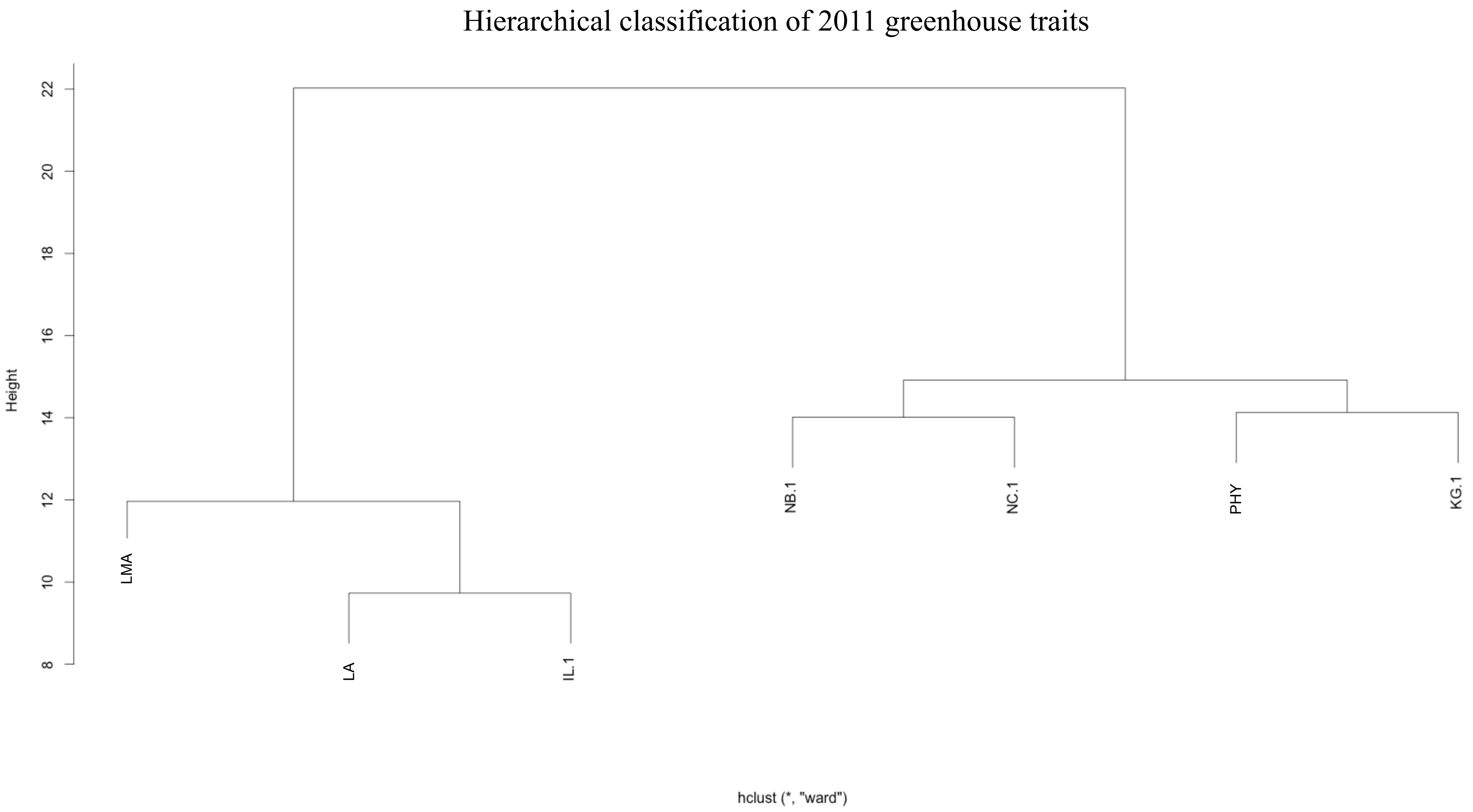

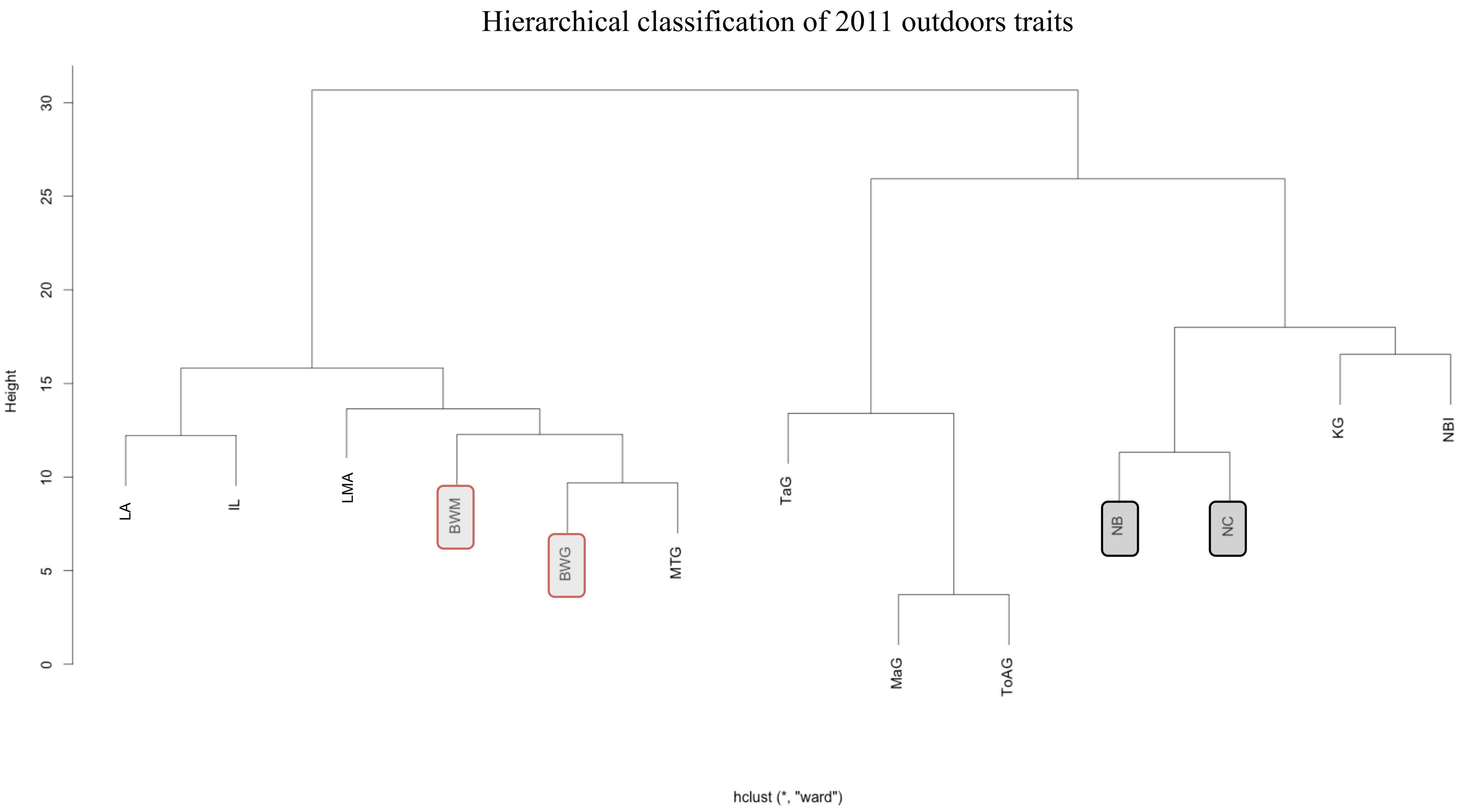

Hierarchical classification of 2011 outdoors traits
Height
0
5
10
15
20
25
30
LA
IL
LMA
BWM
BWG
MTG
TaG
MaG
ToAG
NB
NC
KG
NBI
hclust (*, "ward")

Hierarchical classification of 2012 outdoors traits

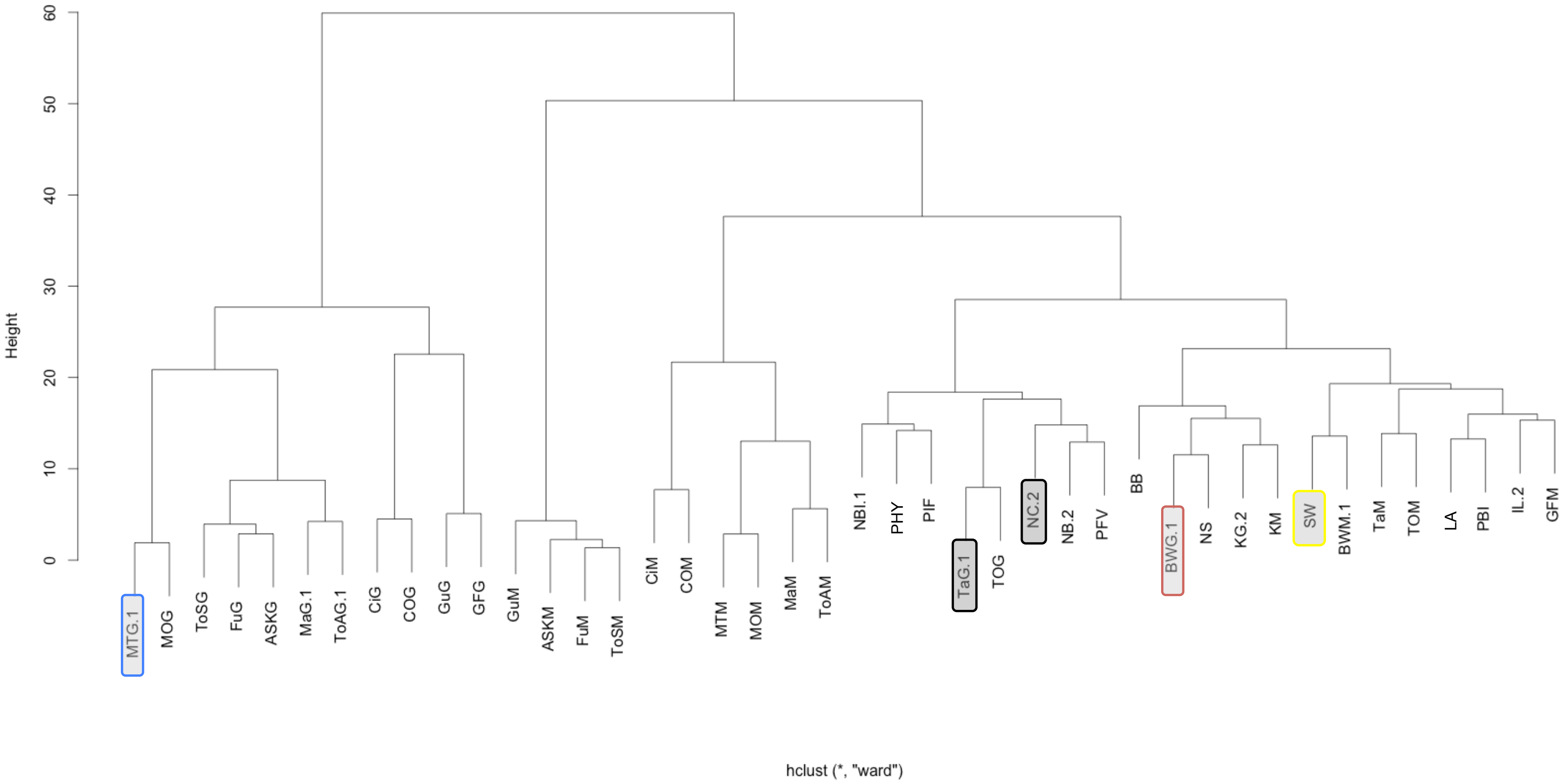

Hierarchical classification of 2013 outdoors traits

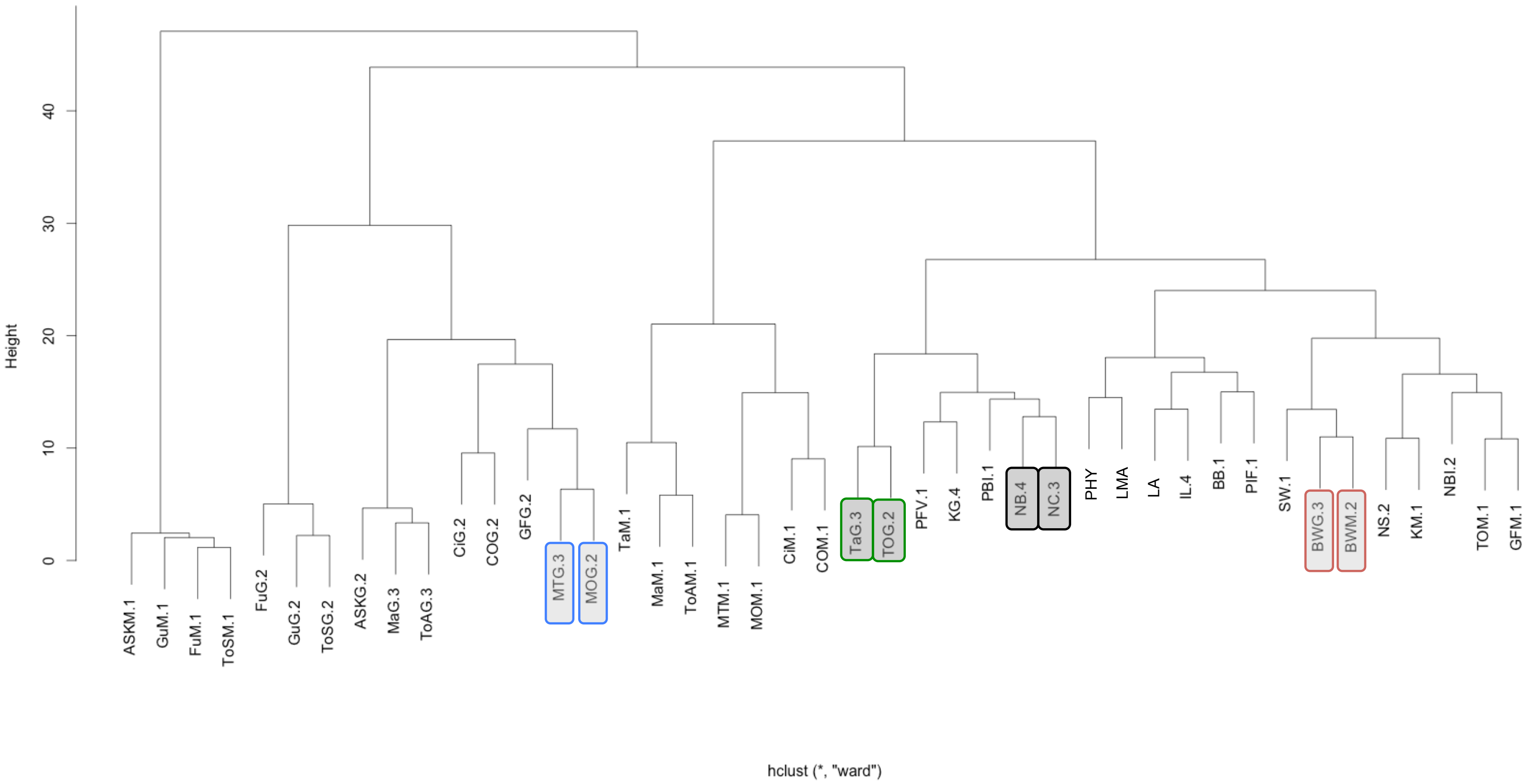

Hierarchical classification of 2014 outdoors traits

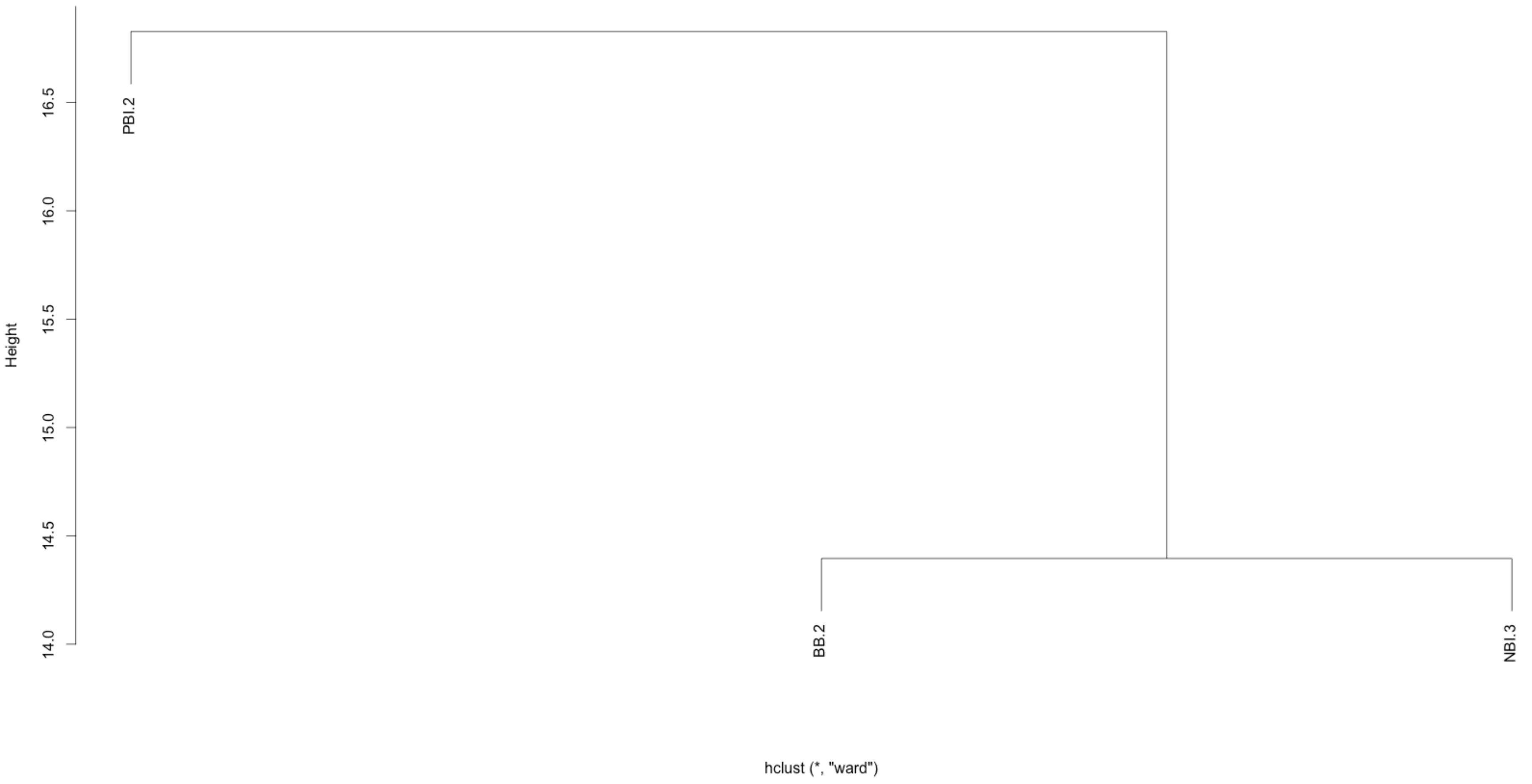

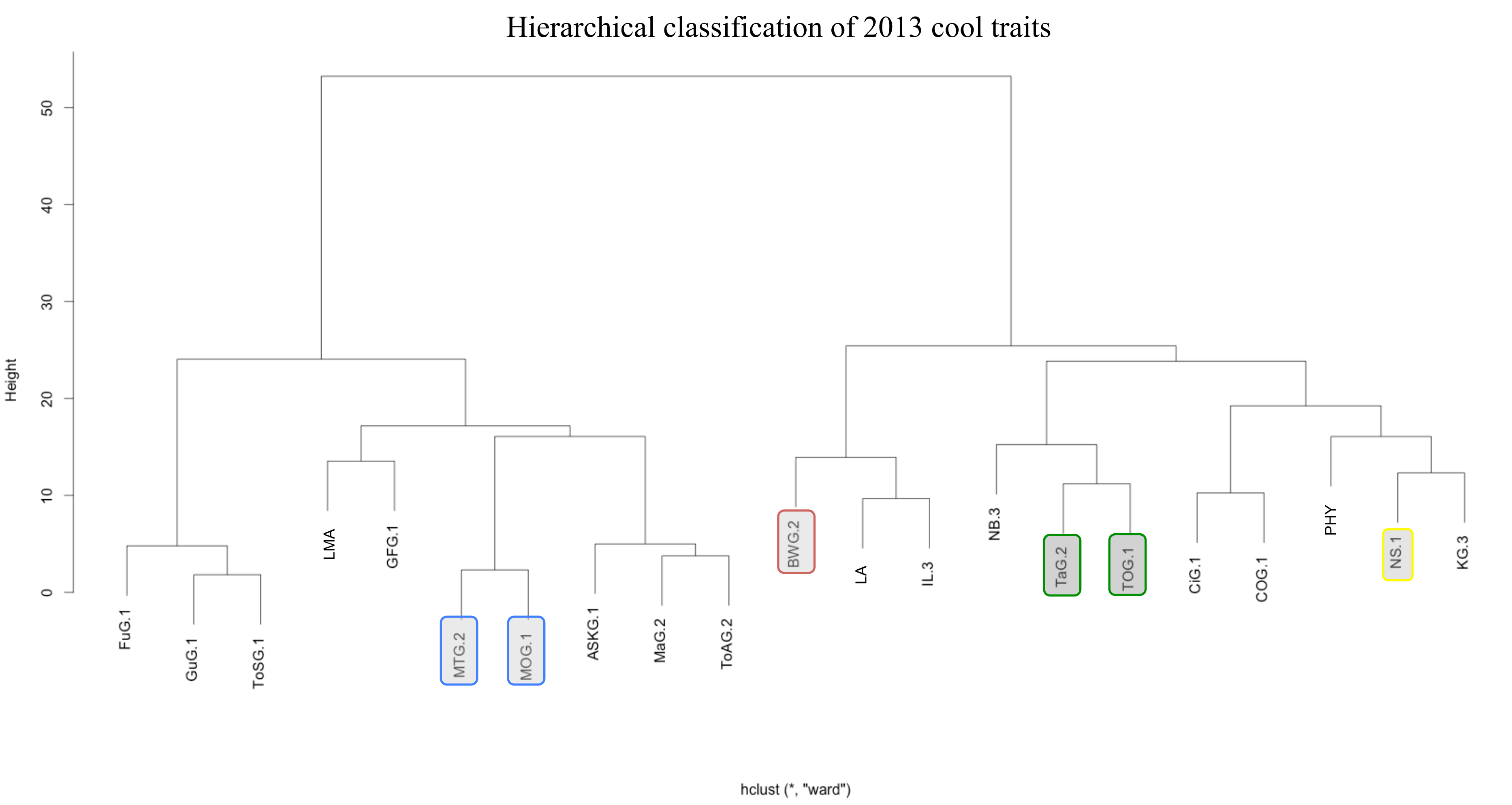

Hierarchical classification of 2013 cool traits
Height
50
40
30
20
10
0
FuG.1
GuG.1
ToSG.1
LMA
GFG.1
MTG.2
MOG.1
ASKG.1
MaG.2
ToAG.2
BWG.2
LA
IL.3
NB.3
TaG.2
TOG.1
CiG.1
COG.1
PHY
NS.1
KG.3
hclust (*, "ward")

Hierarchical classification of 2014 cool traits

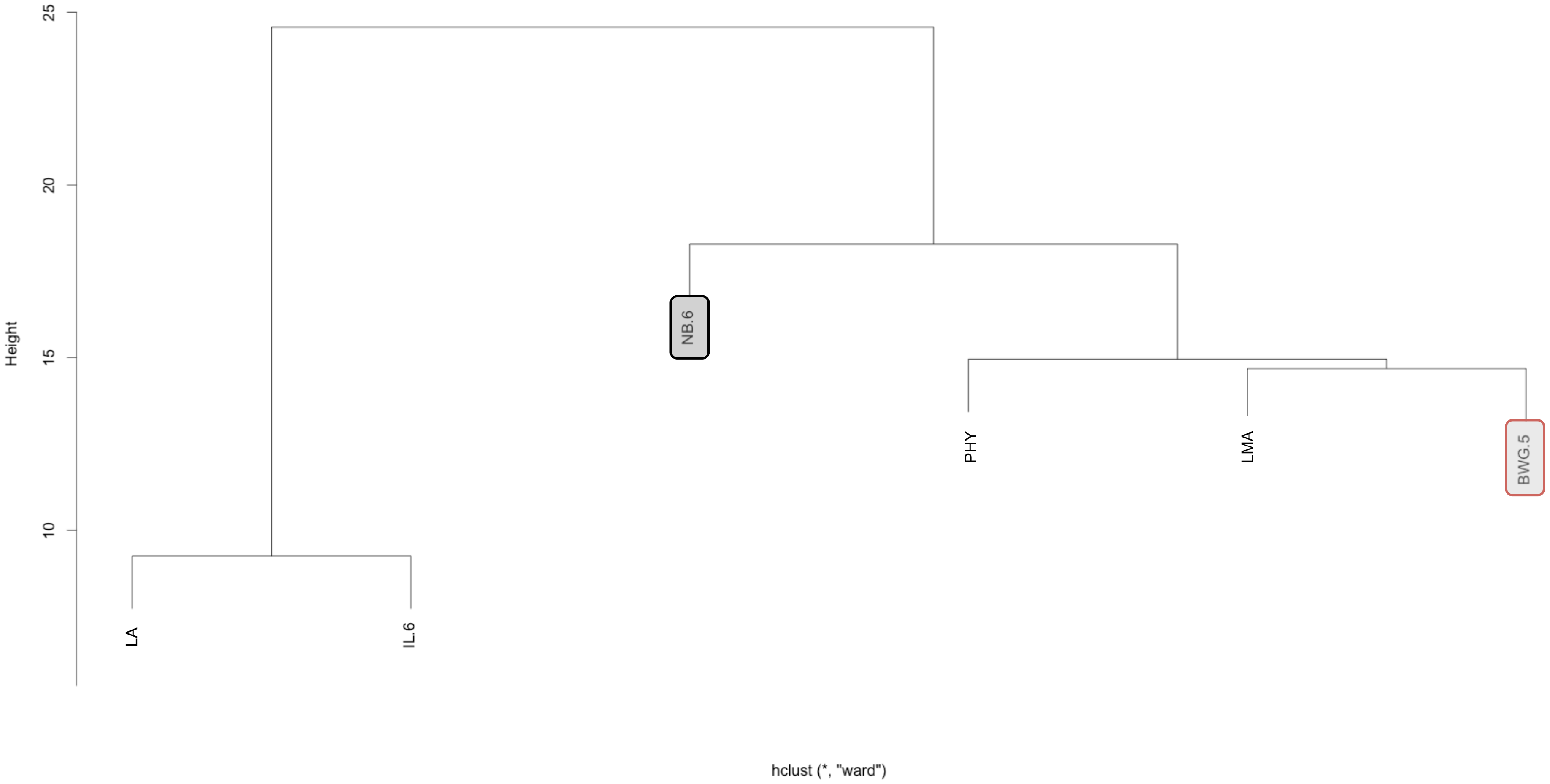

Hierarchical classification of 2013 hot traits

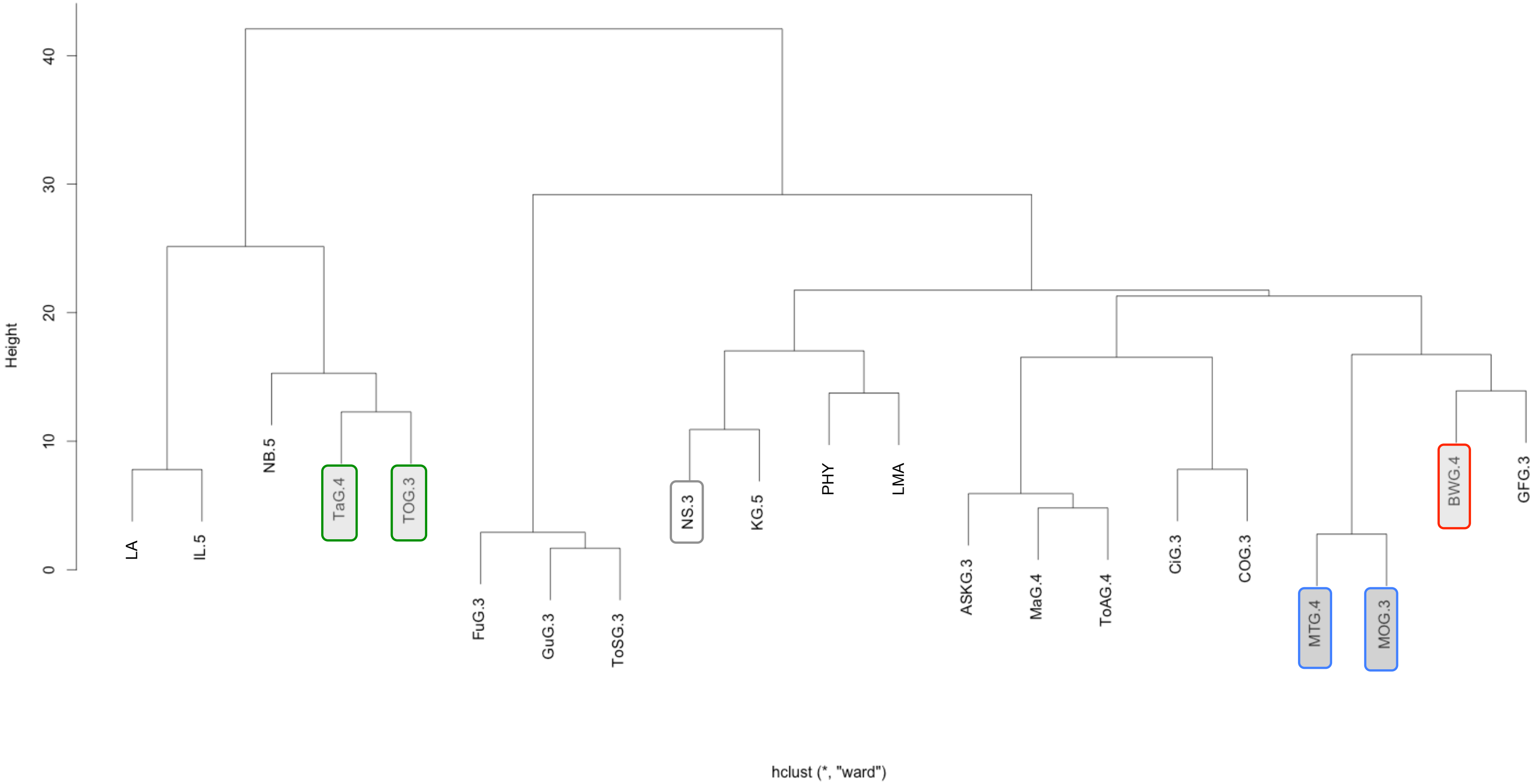

Hierarchical classification of 2014 hot traits

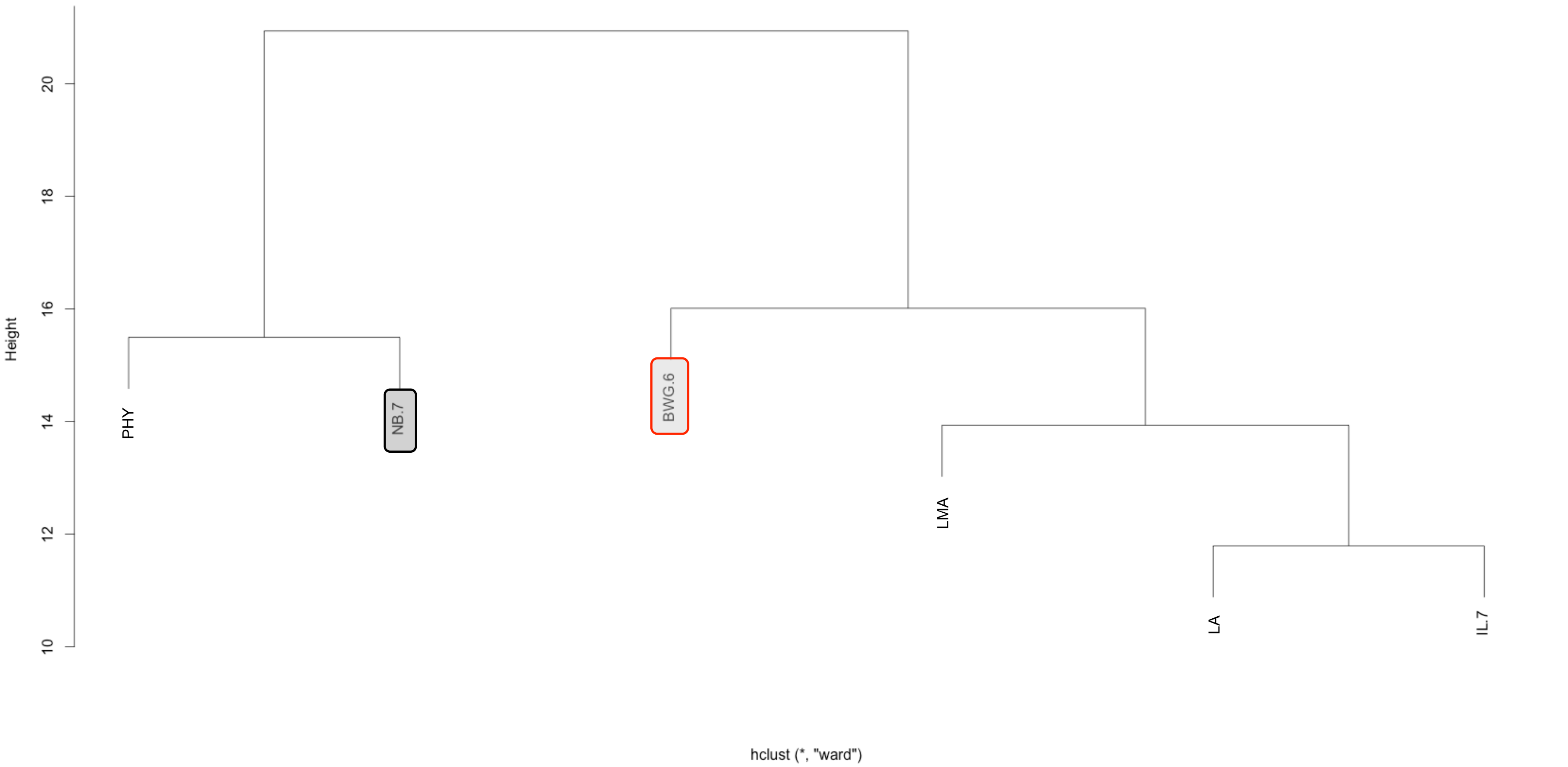

Supplement: Additional file 2: Figure S2. — Hierarchical classification of 43 traits under each growing condition. When two copies of the same traits were measured under the same environment, the mean value of the two copies was used in order to simplify the tree. Different colours represent trait categories for which a repeated QTL was identified. (PDF 616 kb) [file 12870_2015_588_MOESM2_ESM.pdf]
