# Supplementary material for: Identification of stable QTLs for vegetative and reproductive traits in the microvine (Vitis vinifera L.) using the 18 K Infinium chip
Source: BMC Plant Biol. 2015 Aug 19;15:205. doi: 10.1186/s12870-015-0588-0 (PMC4539925; doi:10.1186/s12870-015-0588-0)

Figure S3. Framework parental genetic maps of Picovine and Ugni Blanc *flb* built with SNP markers from the 18K SNP Infinium chip. (A) The Ugni blanc *flb* genetic map. (B) The picovine genetic map.

A

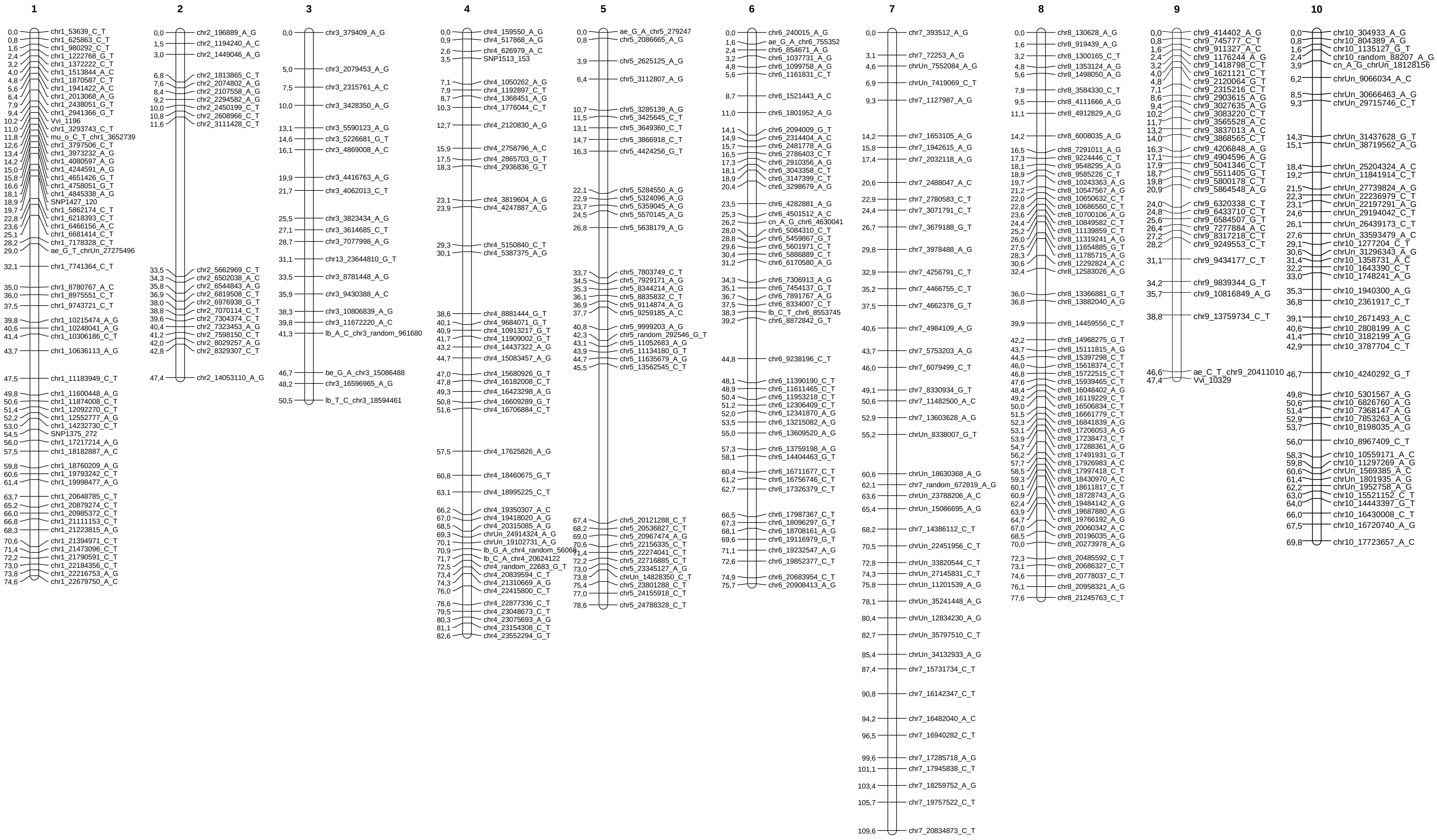

A

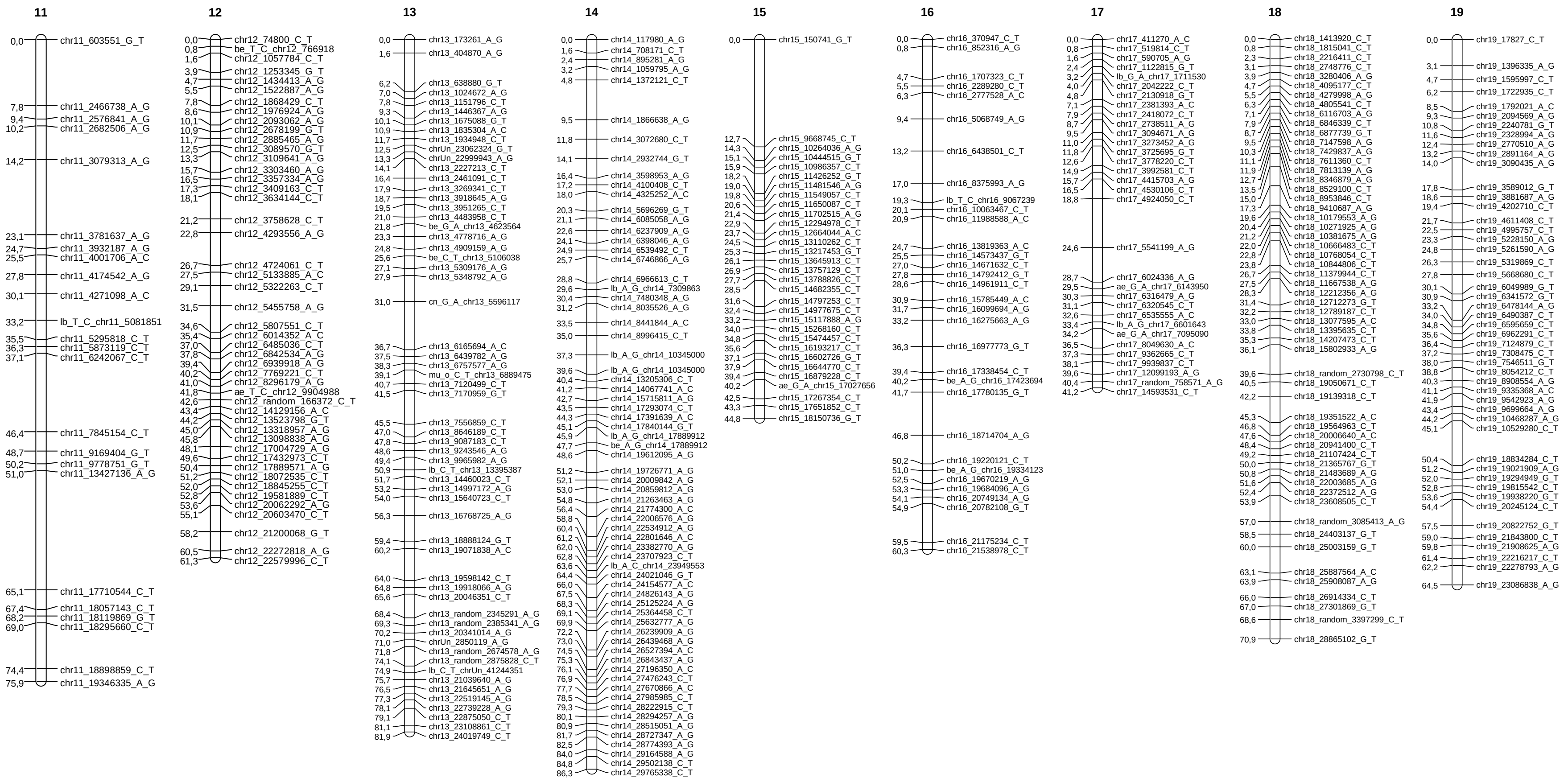

11
0,0 chr11_603551_G_T
7,8 chr11_2466738_A_G
9,4 chr11_2576841_A_G
10,2 chr11_2682506_A_G
14,2 chr11_3079313_A_G
23,1 chr11_3781637_A_G
24,7 chr11_3932187_A_G
25,5 chr11_4001706_A_C
27,8 chr11_4174542_A_G
30,1 chr11_4271098_A_C
33,2 lb_T_C_chr11_5081851
35,5 chr11_5295818_C_T
36,3 chr11_5873119_C_T
37,1 chr11_6242067_C_T
46,4 chr11_7845154_C_T
48,7 chr11_9169404_G_T
50,2 chr11_9778751_G_T
51,0 chr11_13427136_A_G
65,1 chr11_17710544_C_T
67,4 chr11_18057143_C_T
68,2 chr11_18119869_G_T
69,0 chr11_18295660_C_T
74,4 chr11_18898859_C_T
75,9 chr11_19346335_A_G
12
0,0 chr12_74800_C_T
0,8 be_T_C_chr12_766918
1,6 chr12_1057784_C_T
3,9 chr12_1253345_G_T
4,7 chr12_1434413_A_G
5,5 chr12_1522887_A_G
7,8 chr12_1868429_C_T
8,6 chr12_1976924_A_G
10,1 chr12_2093062_A_G
10,9 chr12_2678199_G_T
11,7 chr12_2885465_A_G
12,5 chr12_3089570_G_T
13,3 chr12_3109641_A_G
15,7 chr12_3303460_A_G
16,5 chr12_3357334_A_G
17,3 chr12_3409163_C_T
18,1 chr12_3634144_C_T
21,2 chr12_3758628_C_T
22,8 chr12_4293556_A_G
26,7 chr12_4724061_C_T
27,5 chr12_5133885_A_C
29,1 chr12_5322263_C_T
31,5 chr12_5455758_A_G
34,6 chr12_5807551_C_T
35,4 chr12_6014352_A_C
37,0 chr12_6485036_C_T
37,8 chr12_6842534_A_G
39,4 chr12_6939918_A_G
40,2 chr12_7769221_C_T
41,0 chr12_8296179_A_G
41,8 ae_T_C_chr12_9904988
42,6 chr12_random_166372_C_T
43,4 chr12_14129156_A_C
44,2 chr12_13523798_G_T
45,0 chr12_13318957_A_G
45,8 chr12_13098838_A_G
48,1 chr12_17004729_A_G
49,6 chr12_17432973_C_T
50,4 chr12_17889571_A_G
51,2 chr12_18072535_C_T
52,0 chr12_18845255_C_T
52,8 chr12_19581889_C_T
53,6 chr12_20062292_A_G
55,1 chr12_20603470_C_T
58,2 chr12_21200068_G_T
60,5 chr12_22272818_A_G
61,3 chr12_22579996_C_T
13
0,0 chr13_173261_A_G
1,6 chr13_404870_A_G
6,2 chr13_638880_G_T
7,0 chr13_1024672_A_G
7,8 chr13_1151796_C_T
9,3 chr13_1446367_A_G
10,1 chr13_1675088_G_T
10,9 chr13_1835304_A_C
11,7 chr13_1934948_C_T
12,5 chrUn_23062324_G_T
13,3 chrUn_22999943_A_G
14,1 chr13_2227213_C_T
16,4 chr13_2461091_C_T
17,9 chr13_3269341_C_T
18,7 chr13_3918645_A_G
19,5 chr13_3951265_C_T
21,0 chr13_4483958_C_T
21,8 be_G_A_chr13_4623564
23,3 chr13_4778716_A_G
24,8 chr13_4909159_A_G
25,6 be_C_T_chr13_5106038
27,1 chr13_5309176_A_G
27,9 chr13_5348792_A_G
31,0 cn_G_A_chr13_5596117
36,7 chr13_6165694_A_C
37,5 chr13_6439782_A_G
38,3 chr13_6757577_A_G
39,1 mu_o_C_T_chr13_6889475
40,7 chr13_7120499_C_T
41,5 chr13_7170959_G_T
45,5 chr13_7556859_C_T
47,0 chr13_8646189_C_T
47,8 chr13_9087183_C_T
48,6 chr13_9243546_A_G
49,4 chr13_9965982_A_G
50,9 lb_C_T_chr13_13395387
51,7 chr13_14460023_C_T
53,2 chr13_14997172_A_G
54,0 chr13_15640723_C_T
56,3 chr13_16768725_A_G
59,4 chr13_18888124_G_T
60,2 chr13_19071838_A_C
64,0 chr13_19598142_C_T
64,8 chr13_19918066_A_G
65,6 chr13_20046351_C_T
68,4 chr13_random_2345291_A_G
69,3 chr13_random_2385341_A_G
70,2 chr13_20341014_A_G
71,0 chrUn_2850119_A_G
71,8 chr13_random_2674578_A_G
74,1 chr13_random_2875828_C_T
74,9 lb_C_T_chrUn_41244351
75,7 chr13_21039640_A_G
76,5 chr13_21645651_A_G
77,3 chr13_22519145_A_G
78,1 chr13_22739228_A_G
79,1 chr13_22875050_C_T
81,1 chr13_23108861_C_T
81,9 chr13_24019749_C_T
14
0,0 chr14_117980_A_G
1,6 chr14_708171_C_T
2,4 chr14_895281_A_G
3,2 chr14_1059795_A_G
4,8 chr14_1372121_C_T
9,5 chr14_1866638_A_G
11,8 chr14_3072680_C_T
14,1 chr14_2932744_G_T
16,4 chr14_3598953_A_G
17,2 chr14_4100408_C_T
18,0 chr14_4325252_A_C
20,3 chr14_5696269_G_T
21,1 chr14_6085058_A_G
22,6 chr14_6237909_A_G
24,1 chr14_6398046_A_G
24,9 chr14_6539492_C_T
25,7 chr14_6746866_A_G
28,8 chr14_6966613_C_T
29,6 lb_A_G_chr14_7309863
30,4 chr14_7480348_A_G
31,2 chr14_8035526_A_G
33,5 chr14_8441844_A_C
35,0 chr14_8996415_C_T
37,3 lb_A_G_chr14_10345000
39,6 lb_A_G_chr14_10345000
40,4 chr14_13205306_C_T
41,2 chr14_14067741_A_C
42,7 chr14_15715811_A_G
43,5 chr14_17293074_C_T
44,3 chr14_17391639_A_C
45,1 chr14_17840144_G_T
45,9 lb_A_G_chr14_17889912
47,7 be_A_G_chr14_17889912
48,6 chr14_19612095_A_G
51,2 chr14_19726771_A_G
52,1 chr14_20009842_A_G
53,0 chr14_20859812_A_G
54,8 chr14_21263463_A_G
56,4 chr14_21774300_A_C
58,8 chr14_22006576_A_G
60,4 chr14_22534912_A_G
61,2 chr14_22801646_A_C
62,0 chr14_23382770_A_G
62,8 chr14_23707923_C_T
63,6 lb_A_C_chr14_23949553
64,4 chr14_24021046_G_T
66,0 chr14_24154577_A_C
67,5 chr14_24826143_A_G
68,3 chr14_25125224_A_G
69,1 chr14_25364458_C_T
69,9 chr14_25632777_A_G
72,2 chr14_26239909_A_G
73,0 chr14_26439468_A_G
74,5 chr14_26527394_A_C
75,3 chr14_26843437_A_G
76,1 chr14_27196350_A_C
76,9 chr14_27476243_C_T
77,7 chr14_27670866_A_C
78,5 chr14_27985985_C_T
79,3 chr14_28222915_C_T
80,1 chr14_28294257_A_G
80,9 chr14_28515051_A_G
81,7 chr14_28727347_A_G
82,5 chr14_28774393_A_G
84,0 chr14_29164588_A_G
84,8 chr14_29502138_C_T
86,3 chr14_29765338_C_T
15
0,0 chr15_150741_G_T
12,7 chr15_9668745_C_T
14,3 chr15_10264036_A_G
15,1 chr15_10444515_G_T
15,9 chr15_10986357_C_T
18,2 chr15_11426252_G_T
19,0 chr15_11481546_A_G
19,8 chr15_11549057_C_T
20,6 chr15_11650087_C_T
21,4 chr15_11702515_A_G
22,9 chr15_12294978_C_T
23,7 chr15_12664044_A_C
24,5 chr15_13110262_C_T
25,3 chr15_13217453_G_T
26,1 chr15_13645913_C_T
26,9 chr15_13757129_C_T
27,7 chr15_13788826_C_T
28,5 chr15_14682355_C_T
31,6 chr15_14797253_C_T
32,4 chr15_14977675_C_T
33,2 chr15_15117888_A_G
34,0 chr15_13395635_C_T
34,8 chr15_15474457_C_T
35,6 chr15_15268160_C_T
37,1 chr15_16193217_C_T
37,9 chr15_16602726_G_T
39,4 chr15_16644770_C_T
40,2 chr15_16879228_C_T
ae_G_A_chr15_17027656
42,5 chr15_17267354_C_T
43,3 chr15_17651852_C_T
44,8 chr15_18150736_G_T
16
0,0 chr16_370947_C_T
0,8 chr16_852316_A_G
4,7 chr16_1707323_C_T
5,5 chr16_2289280_C_T
6,3 chr16_2777528_A_C
9,4 chr16_5068749_A_G
13,2 chr16_6438501_C_T
17,0 chr16_8375993_A_G
19,3 lb_T_C_chr16_9067239
20,1 chr16_10063467_C_T
20,9 chr16_11988588_A_C
24,7 chr16_13819363_A_C
25,5 chr16_14573437_G_T
27,0 chr16_14671632_C_T
27,8 chr16_14792412_G_T
28,6 chr16_14961911_C_T
30,9 chr16_15785449_A_C
31,7 chr16_16099694_A_G
33,2 chr16_16275663_A_G
36,3 chr16_16977773_G_T
39,4 chr16_17338454_C_T
40,2 be_A_G_chr16_17423694
41,7 chr16_17780135_G_T
46,8 chr16_18714704_A_G
50,2 chr16_19220121_C_T
51,0 be_A_G_chr16_19334123
52,5 chr16_19670219_A_G
53,3 chr16_19684096_A_G
54,1 chr16_20749134_A_G
54,9 chr16_20782108_G_T
59,5 chr16_21175234_C_T
60,3 chr16_21538978_C_T
17
0,0 chr17_411270_A_C
0,8 chr17_519814_C_T
1,6 chr17_590705_A_G
2,4 chr17_1122815_G_T
3,2 lb_G_A_chr17_1711530
4,0 chr17_2042222_C_T
4,8 chr17_2130918_G_T
7,1 chr17_2381393_A_C
7,9 chr17_2418072_C_T
8,7 chr17_2738511_A_G
9,5 chr17_3094671_A_G
11,0 chr17_3273452_A_G
11,8 chr17_3725695_G_T
12,6 chr17_3778220_C_T
14,9 chr17_3992581_C_T
15,7 chr17_4415703_A_G
16,5 chr17_4530106_C_T
18,8 chr17_4924050_C_T
24,6 chr17_5541199_A_G
28,7 chr17_6024336_A_G
29,5 ae_G_A_chr17_6143950
30,3 chr17_6316479_A_G
31,1 chr17_6320545_C_T
32,6 chr17_6535555_A_C
33,4 lb_A_G_chr17_6601643
34,2 ae_G_A_chr17_7095090
36,5 chr17_8049630_A_C
37,3 chr17_9362665_C_T
38,1 chr17_9939837_C_T
39,6 chr17_12099193_A_G
40,4 chr17_random_758571_A_G
41,2 chr17_14593531_C_T
18
0,0 chr18_1413920_C_T
0,8 chr18_1815041_C_T
2,3 chr18_2216411_C_T
3,1 chr18_2748776_C_T
3,9 chr18_3280406_A_G
4,7 chr18_4095177_C_T
5,5 chr18_4279998_A_G
6,3 chr18_4805541_C_T
7,1 chr18_6116703_A_G
7,9 chr18_6846339_C_T
8,7 chr18_6877739_G_T
9,5 chr18_7147598_A_G
10,3 chr18_7429837_A_G
11,1 chr18_7611360_C_T
11,9 chr18_7813139_A_G
12,7 chr18_8346879_A_G
13,5 chr18_8529100_C_T
15,0 chr18_8953846_C_T
17,3 chr18_9410687_A_G
19,6 chr18_10179553_A_G
20,4 chr18_10271925_A_G
21,2 chr18_10381675_A_G
22,0 chr18_10666483_C_T
22,8 chr18_10768054_C_T
23,8 chr18_10844806_C_T
26,7 chr18_11379944_C_T
27,5 chr18_11667538_A_G
28,3 chr18_12212356_A_G
31,4 chr18_12712273_G_T
32,2 chr18_12789187_C_T
33,0 chr18_13077595_A_C
33,8 chr18_13395635_C_T
35,3 chr18_14207473_C_T
36,1 chr18_15802933_A_G
39,6 chr18_random_2730798_C_T
40,5 chr18_19050671_C_T
42,2 chr18_19139318_C_T
45,3 chr18_19351522_A_C
46,8 chr18_19564963_C_T
47,6 chr18_20006640_A_C
48,4 chr18_20941400_C_T
49,2 chr18_21107424_C_T
50,0 chr18_21365767_G_T
50,8 chr18_21483689_A_G
51,6 chr18_22003685_A_G
52,4 chr18_22372512_A_G
53,9 chr18_23608505_C_T
57,0 chr18_random_3085413_A_G
58,5 chr18_24403137_G_T
60,0 chr18_25003159_G_T
63,1 chr18_25887564_A_C
63,9 chr18_25908087_A_G
66,0 chr18_26914334_C_T
67,0 chr18_27301869_G_T
68,6 chr18_random_3397299_C_T
70,9 chr18_28865102_G_T
19
0,0 chr19_17827_C_T
3,1 chr19_1396335_A_G
4,7 chr19_1595997_C_T
6,2 chr19_1722935_C_T
8,5 chr19_1792021_A_C
9,3 chr19_2094569_A_G
10,8 chr19_2240781_G_T
11,6 chr19_2328994_A_G
12,4 chr19_2770510_A_G
13,2 chr19_2891164_A_G
14,0 chr19_3090435_A_G
17,8 chr19_3589012_G_T
18,6 chr19_3881687_A_G
19,4 chr19_4202710_C_T
21,7 chr19_4611408_C_T
22,5 chr19_4995757_C_T
23,3 chr19_5228150_A_G
24,8 chr19_5261590_A_G
26,3 chr19_5319869_C_T
27,8 chr19_5668680_C_T
30,1 chr19_6049989_G_T
30,9 chr19_6341572_G_T
33,2 chr19_6478144_A_G
34,0 chr19_6490387_C_T
34,8 chr19_6595659_C_T
35,6 chr19_6962291_C_T
36,4 chr19_7124879_C_T
37,2 chr19_7308475_C_T
38,0 chr19_7546511_G_T
38,8 chr19_8054212_C_T
40,3 chr19_8908554_A_G
41,1 chr19_9335368_A_C
41,9 chr19_9542923_A_G
43,4 chr19_9699664_A_G
44,2 chr19_10468287_A_G
45,1 chr19_10529280_C_T
50,4 chr19_18834284_C_T
51,2 chr19_19021909_A_G
52,0 chr19_19294949_G_T
52,8 chr19_19815542_C_T
53,6 chr19_19938220_G_T
54,4 chr19_20245124_C_T
57,5 chr19_20822752_G_T
59,0 chr19_21843800_C_T
59,8 chr19_21908625_A_G
61,4 chr19_22216217_C_T
62,2 chr19_22278793_A_G
64,5 chr19_23086838_A_G

B

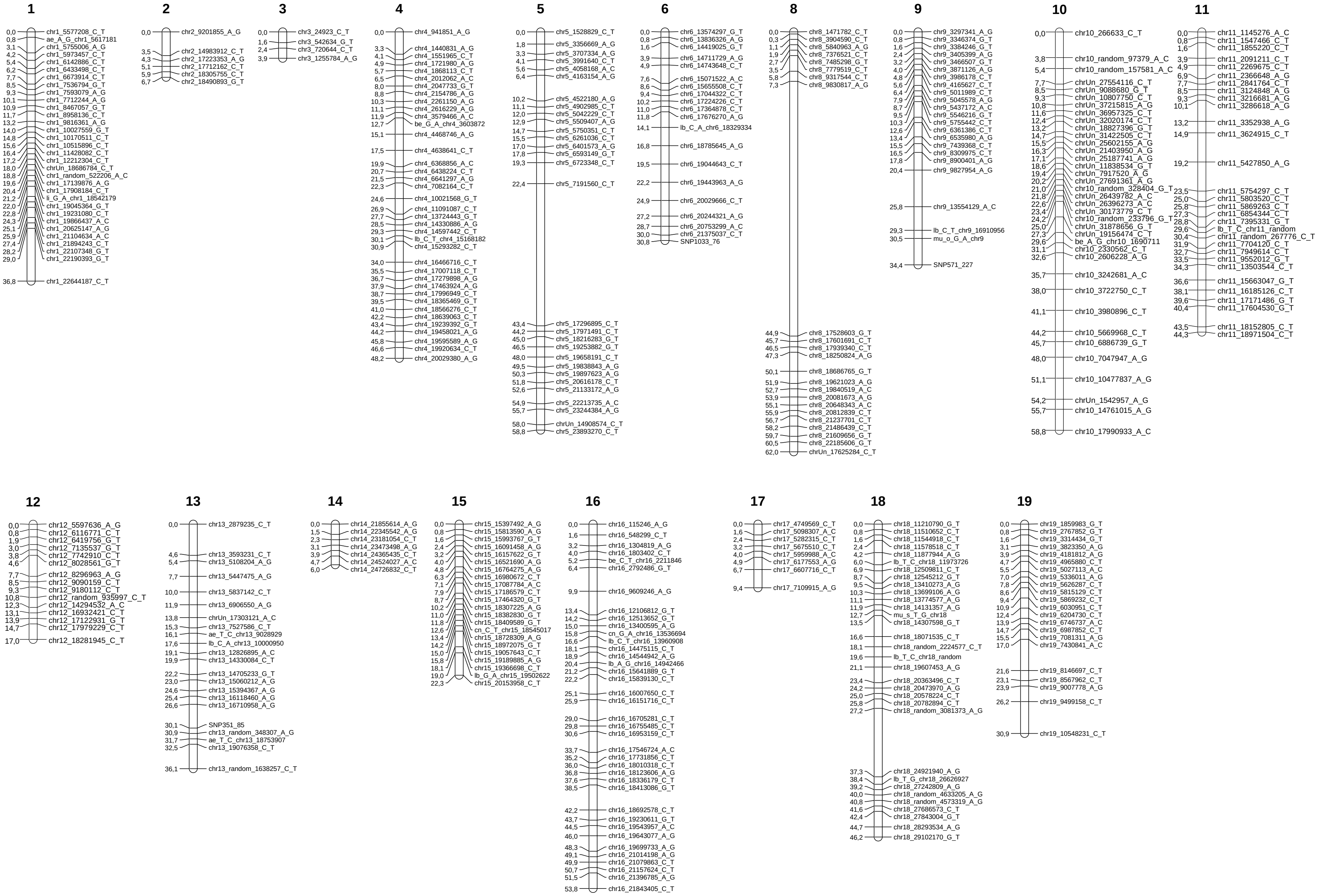

Supplement: Additional file 4: Figure S3 — Framework parental genetic maps of Picovine and Ugni Blanc flb built with SNP markers from the 18 K SNP Infinium chip. (A) The Ugni Blanc flb genetic map. (B) The Picovine genetic map. (PDF 368 kb) [file 12870_2015_588_MOESM4_ESM.pdf]
