# Supplementary material for: Identification of stable QTLs for vegetative and reproductive traits in the microvine (Vitis vinifera L.) using the 18 K Infinium chip
Source: BMC Plant Biol. 2015 Aug 19;15:205. doi: 10.1186/s12870-015-0588-0 (PMC4539925; doi:10.1186/s12870-015-0588-0)

Figure S4. SNP positions on genetic maps as a function of their physical position on the reference genome version 12X.2. The maternal parent is the Picovine (blue circles) and the paternal parent is the Ugni Blanc *flb* (pink circles).

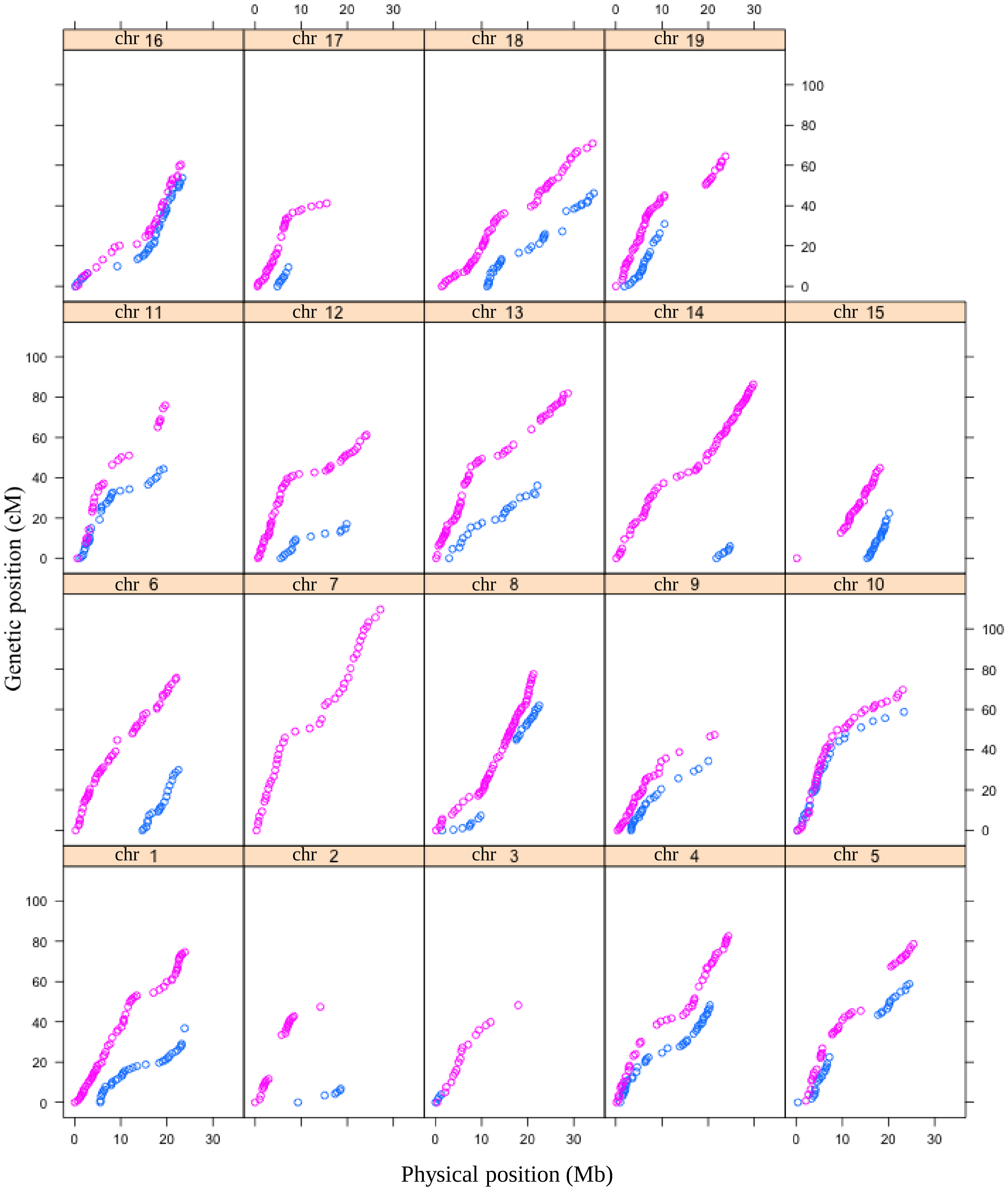

Supplement: Additional file 6: Figure S4. — SNP positions on genetic maps as a function of their physical position on the reference genome version 12X.2. The maternal parent is the Picovine (blue circles) and the paternal parent is the Ugni Blanc flb (pink circles). (PDF 321 kb) [file 12870_2015_588_MOESM6_ESM.pdf]
