# Supplementary material for: Identification of stable QTLs for vegetative and reproductive traits in the microvine (Vitis vinifera L.) using the 18 K Infinium chip
Source: BMC Plant Biol. 2015 Aug 19;15:205. doi: 10.1186/s12870-015-0588-0 (PMC4539925; doi:10.1186/s12870-015-0588-0)
Supplement: Additional file 10: Figure S5. — Distribution of the number of candidate genes expressed in appropriate organs, according to the main biological functions related to the repeated QTLs identified. (PDF 187 kb) [file 12870_2015_588_MOESM10_ESM.pdf]

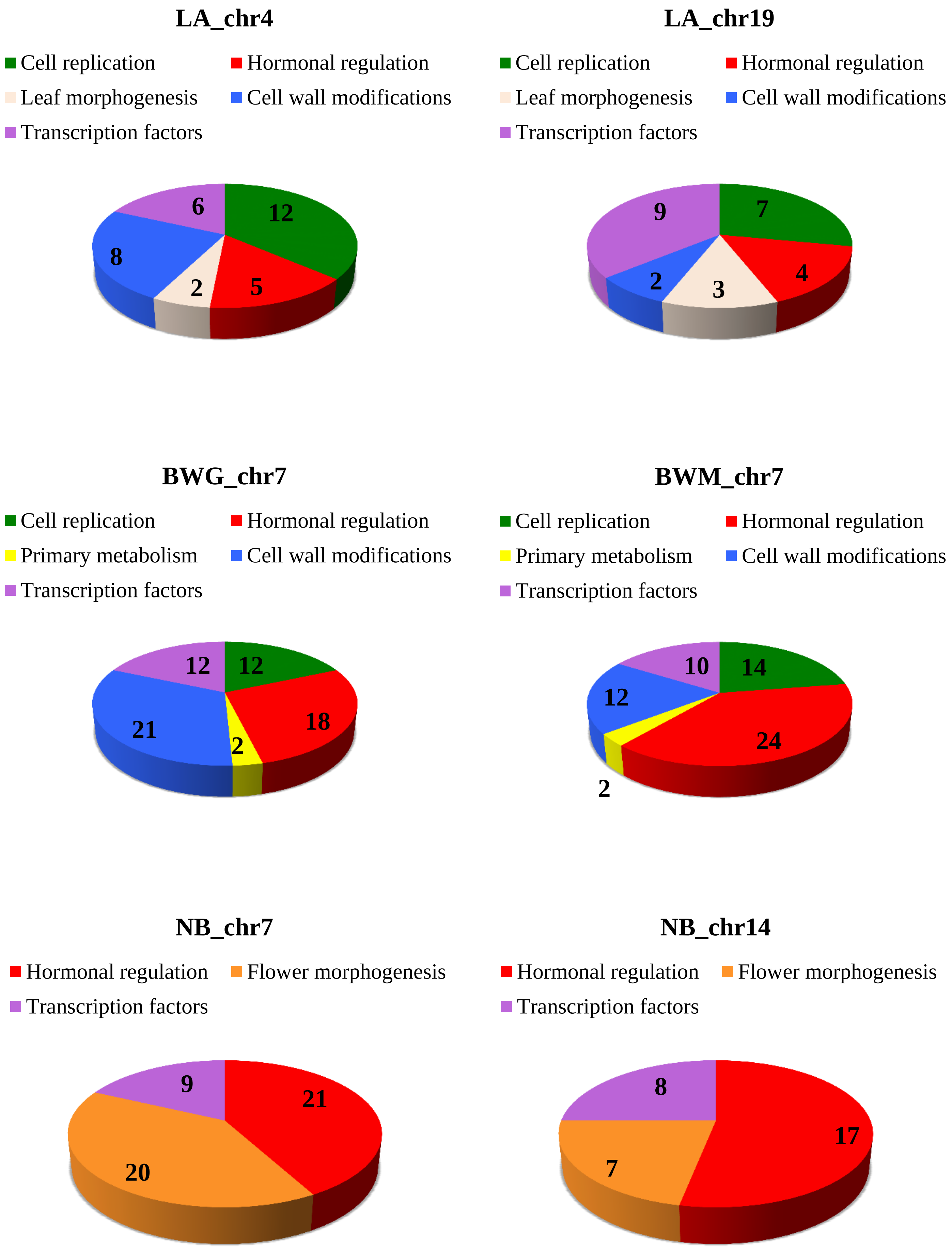
LA_chr4
Cell replication
Hormonal regulation
Leaf morphogenesis
Cell wall modifications
Transcription factors
6
12
8
2
5
LA_chr19
Cell replication
Hormonal regulation
Leaf morphogenesis
Cell wall modifications
Transcription factors
9
7
2
3
4
BWG_chr7
Cell replication
Hormonal regulation
Primary metabolism
Cell wall modifications
Transcription factors
12
12
18
21
2
BWM_chr7
Cell replication
Hormonal regulation
Primary metabolism
Cell wall modifications
Transcription factors
10
14
12
24
2
NB_chr7
Hormonal regulation
Flower morphogenesis
Transcription factors
9
21
20
NB_chr14
Hormonal regulation
Flower morphogenesis
Transcription factors
8
17
7

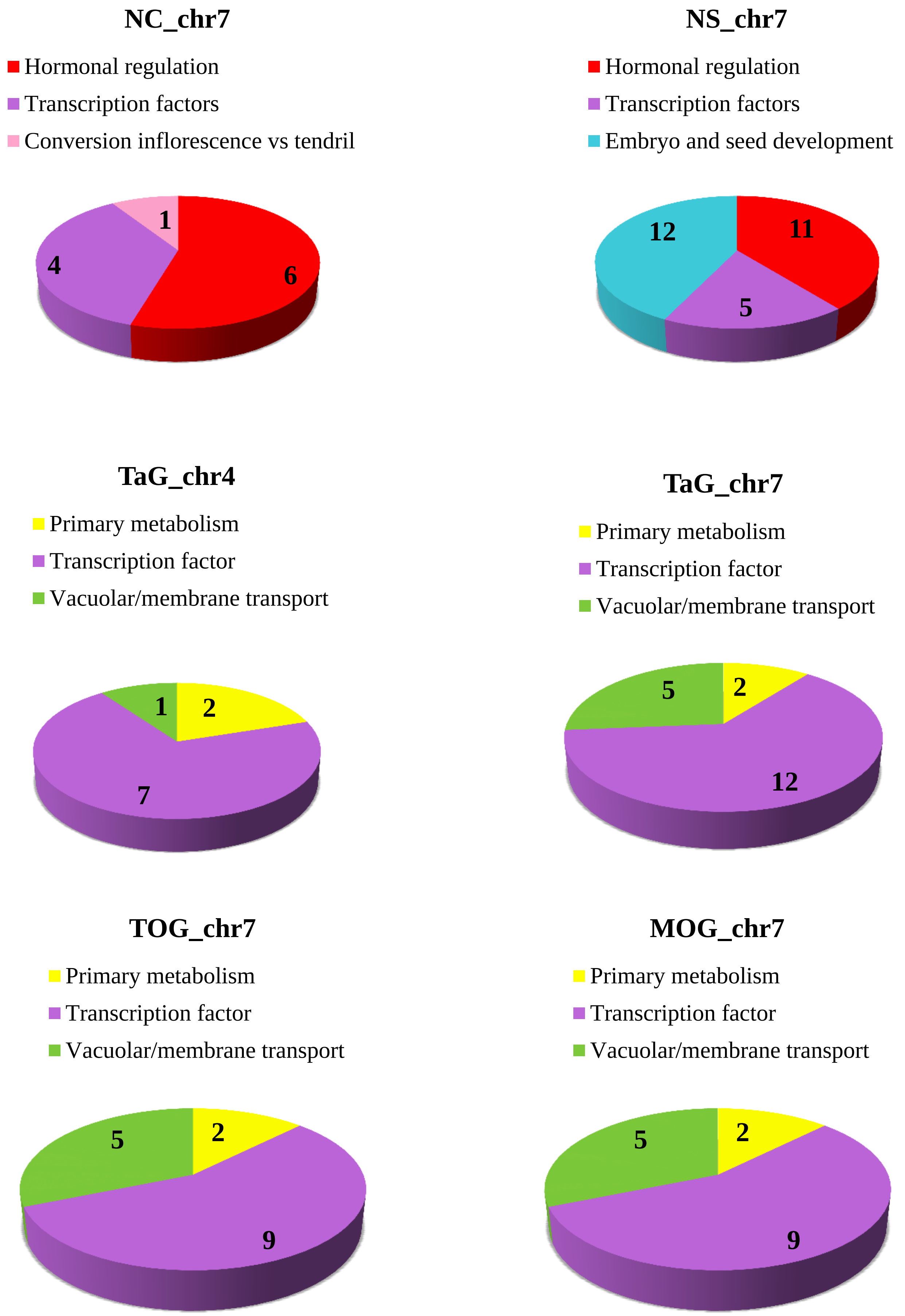
NC_chr7
Hormonal regulation
Transcription factors
Conversion inflorescence vs tendril
1
4
6
NS_chr7
Hormonal regulation
Transcription factors
Embryo and seed development
12
11
5
TaG_chr4
Primary metabolism
Transcription factor
Vacuolar/membrane transport
1
2
7
TaG_chr7
Primary metabolism
Transcription factor
Vacuolar/membrane transport
5
2
12
TOG_chr7
Primary metabolism
Transcription factor
Vacuolar/membrane transport
5
2
9
MOG_chr7
Primary metabolism
Transcription factor
Vacuolar/membrane transport
5
2
9

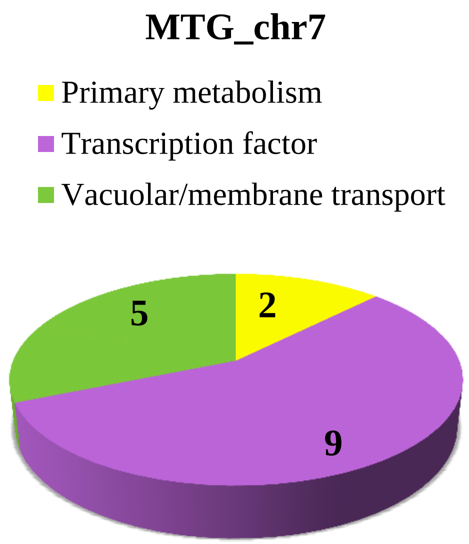
MTG_chr7
Primary metabolism
Transcription factor
Vacuolar/membrane transport
5
2
9

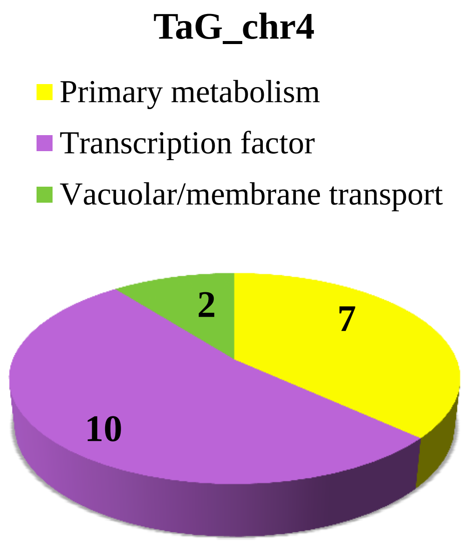
TaG_chr4
Primary metabolism
Transcription factor
Vacuolar/membrane transport
2
7
10
